# Effects of antiplatelet therapy on stroke risk by brain imaging features of intracerebral haemorrhage and cerebral small vessel diseases: subgroup analyses of the RESTART randomised, open-label trial

**DOI:** 10.1016/S1474-4422(19)30184-X

**Published:** 2019-07

**Authors:** Rustam Al-Shahi Salman, David P Minks, Dipayan Mitra, Mark A Rodrigues, Priya Bhatnagar, Johann C du Plessis, Yogish Joshi, Martin S Dennis, Gordon D Murray, David E Newby, Peter A G Sandercock, Nikola Sprigg, Jacqueline Stephen, Cathie L M Sudlow, David J Werring, William N Whiteley, Joanna M Wardlaw, Philip M White, RESTART Collaboration, Colin Baigent, Daniel Lasserson, Frank Sullivan, Johanna Carrie, Javier Rojas, Shannon Amoils, John Bamford, Jane Armitage, Gabriel Rinkel, Gordon Lowe, Jonathan Emberson, Karen Innes, Lynn Dinsmore, Jonathan Drever, Carol Williams, David Perry, Connor McGill, David Buchanan, Allan Walker, Aidan Hutchison, Christopher Matthews, Ruth Fraser, Aileen McGrath, Ann Deary, Rosemary Anderson, Pauli Walker, Christian Hansen, Richard Parker, Aryelly Rodriguez, Malcolm Macleod, Thomas Gattringer, Jeb Palmer, Eleni Sakka, Jennifer Adil-Smith, David Minks, Dipayan Mitra, Priya Bhatnagar, Johannes du Plessis, Yogish Joshi, Christine Lerpiniere, Richard O'Brien, Seona Burgess, Gillian Mead, Ruth Paulton, Fergus Doubal, Katrina McCormick, Neil Hunter, Pat Taylor, Ruwan Parakramawansha, Jack Perry, Gordon Blair, Allan MacRaild, Adrian Parry-Jones, Mary Johnes, Stephanie Lee, Kelly Marie Shaw, Ilse Burger, Martin Punter, Andrea Ingham, Jane Perez, Zin Naing, Jordi Morell, Tracy Marsden, Andrea Hall, Sally Marshall, Louise Harrison, Rowilson Jarapa, Edith Wood, Victoria O'Loughlin, David Cohen, Silvie Davies, Kelechi Njoku, Mushiya Mpelembue, Laura Burgess, Radim Licenik, Mmua Ngwako, Nabeela Nisar, Rangah Niranchanan, Tatjana Roganova, Rajaram Bathula, Joseph Devine, Anette David, Anne Oshodi, Fenglin Guo, Emmanuelle Owoyele, Varthi Sukdeo, Robert Ballantine, Mudhar Abbdul-saheb, Angela Chamberlain, Aberami Chandrakumar, Philip Poku, Kirsty Harkness, Catrin Blank, Emma Richards, Ali Ali, Faith Kibutu, Olesia Balitska, Kathryn Birchall, Pauline Bayliss, Clare Doyle, Kathy Stocks, Arshad Majis, Jo Howe, Christine Kamara, Luke Barron, Ahmad Maatouk, Ralf Lindert, Katy Dakin, Jessica Redgrave, Biju Bhaskaran, Isam Salih, Debs Kelly, Susan Szabo, Dawn Tomlin, Helen Bearne, Jean Buxton, Pauline Fitzell, Georgina Ayres, Afaq Saulat, Kathleen Horan, Joanne Garfield-Smith, Harbens Bhakri, Paul Guyler, Devesh Sinha, Thayalini Loganathan, Amber Siddiqui, Anwer Siddiqui, Lucy Coward, Swapna Kunhunny, Sharon Tysoe, Rajalakshmi Orath Prabakaran, Shyam Kelavkar, Sindhu Rashmi, David Ngo, Kheng Xiong Ng, Nisha Menon, Sweni Shah, Mark Barber, Derek Esson, Fiona Brodie, Talat Anjum, Mushtaq Wani, Manju Krishnan, Leanne Quinn, Jayne Spencer, Terry Jones, Helen Thompson-Jones, Lynne Dacey, Srikanth Chenna, Sharon Storton, Sarah Thomas, Teresa Beaty, Shelley Treadwell, Caroline Davies, Susan Tucker, Lynda Connor, Peter Slade, Glyn Gainard, Girish Muddegowda, Ranjan Sanyal, Alda Remegoso, Nenette Abano, Chelsea Causley, Racquel Carpio, Stephanie Stevens, Adrian Butler, Resti Varquez, Hayley Denic, Francis Alipio, Andrew Moores, Adrian Barry, Holly Maguire, Jeanette Grocott, Kay Finney, Sue Lyjko, Christine Roffe, Joanne Hiden, Phillip Ferdinand, Vera Cvoro, Khalil Ullah, Nicola Chapman, Mandy Couser, Susan Pound, Katrina McCormick, Sean Mcauley, Senthil Raghunathan, Faye Shelton, Amanda Hedstrom, Margi Godfrey, Diane Havard, Amanda Buck, Kailash Krishnan, Nicola Gilzeane, Jack Roffe, Judith Clarke, Katherine Whittamore, Saima Sheikh, Nikola Sprigg, Rekha Keshvara, Carla Jordan, Benjamin Jackson, Gwendoline Wilkes, Jason Appleton, Zhe Law, Oliver Matias, Evangelos Vasileiadis, Cathy Mason, Anthea Parry, Geraldine Landers, Melinda Holden, Basaam Aweid, Khalid Rashed, Linda Balian, Carinna Vickers, Elizabeth Keeling, Sarah Board, Joanna Allison, Clare Buckley, Barbara Williams-Yesson, Joanne Board, Tressy Pitt-Kerby, Alfonso Tanate, Diane Wood, Manohar Kini, Dinesh Chadha, Deborah Walstow, Rosanna Fong, Robert Luder, Tolu Adesina, Jill Gallagher, Hayley Bridger, Elodie Murali, Maneesh Bhargava, Chloe van Someren, Frances Harrington, Abhijit Mate, Ali James, Gillian Courtauld, Christine Schofield, Katja Adie, Linda Lucas, Kirsty Bond, Bev Maund, Sam Ellis, Paul Mudd, Martin James, Samantha Keenan, Angela Bowring, Julie Cageao, Hayley Kingwell, Caroline Roughan, Anthony Hemsley, Jane Sword, David Strain, Keniesha Miller, Anita Goff, Karin Gupwell, Kevin Thorpe, Hedley Emsley, Shuja Punekar, Alison McLoughlin, Sulaiman Sultan, Bindu Gregory, Sonia Raj, Donna Doyle, Keith Muir, Wilma Smith, Angela Welch, Fiona Moreton, Bharath Kumar Cheripelli, Salwa El Tawil, Dheeraj Kalladka, Xuya Huang, Nicola Day, Sankaranarayanan Ramachandran, Caroline Crosbie, Jennifer Elliot, Tony Rudd, Katherine Marks, Ajay Bhalla, Jonathan Birns, Sagal Kullane, Nic Weir, Christopher Allen, Vanessa Pressly, Pam Crawford, Emma Battersby-Wood, Alex Blades, Shuna Egerton, Ashleigh Walters, Sue Evans, James Richard Marigold, Fiona Smith, Gabriella Howard, Imogen Gartrell, Simon Smith, Robyn Creeden, Chloe Cox, Cherish Boxall, Jonathan Hewitt, Claire Nott, Procter Sarah, Jessica Whiteman, Steve Buckle, Rebecca Wallace, Rina Mardania, Jane Gray, Claire Triscott, Anand Nair, Jill Greig, Pratap Rana, Matthew Robinson, Mohammad Irfan Alam, David Werring, Duncan Wilson, Caroline Watchurst, Maria Brezitski, Luci Crook, Ifan Jones, Azra Banaras, Krishna Patel, Renuka Erande, Caroline Hogan, Isabel Hostettler, Amy Ashton, Shez Feerick, Nina Francia, Nnebuife Oji, Emma Elliott, Talal Al-Mayhani, Martin Dennis, Cathie Sudlow, William Whiteley, Christine Lerpiniere, Rustam Al-Shahi Salman, Ruth Fraser, Dipankar Dutta, Pauline Brown, Deborah Ward, Fiona Davis, Jennifer Turfrey, Chloe Hughes, Kayleigh Collins, Rehana Bakawala, Susan O'Connell, Jon Glass, David Broughton, Dinesh Tryambake, Lynn Dixon, Kath Chapman, Andrew Young, Adrian Bergin, Andrew Sigsworth, Aravind Manoj, Glyn Fletcher, Paula Lopez, Penelope Cox, Mark Wilkinson, Paul Fitzsimmons, Nikhil Sharma, James Choulerton, Denise Button, Lindsey Dow, Lukuman Gbadamoshi, Joanne Avis, Barbara Madigan, Stephanie McCann, Louise Shaw, Deborah Howcroft, Suzanne Lucas, Andrew Stone, Gillian Cluckie, Caroline Lovelock, Brian Clarke, Neha Chopra, Natasha Clarke, Bhavini Patel, Kate Kennedy, Rebecca Williams, Adrian Blight, Joanna O'Reilly, Chukwuka Orefo, Nilofer Dayal, Rita Ghatala, Temi Adedoyin, Fran Watson, Sarah Trippier, Lillian Choy, Barry Moynihan, Usman Khan, Val Jones, Naomi Jeyaraj, Lourda Kerin, Kamy Thavanesan, Divya Tiwari, Chantel Cox, Anja Ljubez, Laura Tucker, Arshi Iqbal, Caroline Bagnall, Marketa Keltos, Josh Roberts, Becky Jupp, Catherine Ovington, Emily Rogers, Owen David, Jo Bell, Barbara Longland, Gail Hann, Martin Cooper, Mohammad Nasar, Anoja Rajapakse, Inez Wynter, Ijaz Anwar, Helen Skinner, Tarn Nozedar, Damian McArdle, Balakrishna Kumar, Susan Crawford, Arunkumar Annamalai, Alex Ramshaw, Clare Holmes, Sarah Caine, Mairead Osborn, Emily Dodd, Peter Murphy, Nicola Devitt, Pauline Baker, Amy Steele, Lucy Belle Guthrie, Samantha Clarke, Ahamad Hassan, Dean Waugh, Emelda Veraque, Linetty Makawa, Mary Kambafwile, Marc Randall, Vasileios Papavasileiou, Claire Cullen, Jenny Peters, Hlaing Thant, Tanya Ingram, Mellor Zoe, Ramesh Durairaj, Melanie Harrison, Sarah Stevenson, Daniela Shackcloth, Jordan Ewing, Victoria Sutton, Mark McCarron, Jacqueline McKee, Mandy Doherty, Ferghal McVerry, Caroline Blair, Mary MacLeod, Janice Irvine, Heather Gow, Jacqueline Furnace, Anu Joyson, Baljit Jagpal, Sarah Ross, Katrina Klaasen, Sandra Nelson, Rebecca Clarke, Nichola Crouch, Beverly MacLennan, Vicky Taylor, Daniel Epstein, Ifan Jones, Avani Shukla, Vinodh Krishnamurthy, Paul Nicholas, Sammie Qureshi, Adam Webber, Justin Penge, Hawraman Ramadan, Stuart Maguire, Chris Patterson, Ruth Bellfield, Brigid Hairsine, Kelvin Stewart, Michaela Hooley, Outi Quinn, Bella Richard, Sally Moseley, Claire Nott, Steve Buckle, Procter Sarah, Jessica Whiteman, Mandy Edwards, Heidi Lawson, Rebecca Wallace, Claire Triscott, Michelle Tayler, Yogish Pai, Mahesh Dhakal, Bernard Esisi, Sofia Dima, Gemma Marie Smith, Mark Garside, Muhammad Naeem, Vidya Baliga, Gill Rogers, Ellen Brown, David Bruce, Rachel Hayman, Susan Clayton, Ed Gamble, Rebecca Grue, Bethan Charles, Adam Hague, Sujata Blane, Caroline Lambert, Afnan Chaudhry, Thomas Harrison, Kari Saastamoinen, Dionne Hove, Laura Howaniec, Gemma Grimwood, Ozlem Redjep, Fiona Humphries, Lucia Argandona, Larissa Cuenoud, Esther Erumere, Sageet Amlani, Grace Auld, Afraim Salek-Haddadi, Ursula Schulz, James Kennedy, Gary Ford, Philip Mathieson, Ian Reckless, Rachel Teal, Giulia Lenti, George Harston, Eoin O'Brien, Joanne Mcgee, Jennifer Mitchell, Elaine Amis, Dominic Handley, Siobhan Kelly, George Zachariah, Jobbin Francis, Sarah Crisp, Juliana Sesay, Sarah Finlay, Helen Hayhoe, Niamh Hannon, Tom Hughes, Bethan Morse, Henry De Berker, Emma Tallantyre, Ahmed Osman, Susan White, Stefan Schwarz, Benjamin Jelley, Rajendra Yadava, Khalid Azhar, Julie Reddan, Mirriam Sangombe, Samantha Stafford, Ken Fotherby, Debbie Morgan, Farrukh Baig, Karla Jennings-Preece, Donna Butler, Nasar Ahmad, Angela Willberry, Angela Stevens, Baljinder Rai, Prasad Siddegowda, Peter Howard, Afaq Saulat, Lisa Hyatt, Tracey Dobson, David Jarrett, Suheil Ponnambath, Jane Tandy, Yasmin Harrington-Davies, Rebecca Butler, Claire James, Stacey Valentine, Anne Suttling, Peter Langhorne, Gillian Kerr, Fiona Wright, Ruth Graham, Christine McAlpine, Mohammad Shahzad Iqbal, Louise Humphreys, Kath Pasco, Olga Balazikova, Ashraf Nasim, Cassilda Peixoto, Louise Gallagher, Shahrzad Shahmehri, Sandip Ghosh, Elizabeth Barrie, Danielle Gilmour, Margo Henry, Tom Webb, Linda Cowie, Hannah Rudenko, Shanni McDonald, Natasha Schumacher, Susannah Walker, Tracey Cosier, Anna Verrion, Eva Beranova, Audrey Thomson, Marius Venter, Arindam Kar, Sheila Mashate, Kirsten Harvey, Léjeune Gardener, Vinh Nguyen, Omid Halse, Olivia Geraghty, Beth Hazel, Peter Wilding, Victoria Tilley, Bernard Esisi, Tim Cassidy, Beverley McClelland, Maria Bokhari, Timothy England, Amanda Hedstrom, Mohana Maddula, Richard Donnelly, Paul Findlay, Ashish Macaden, Ian Shread, Charlotte Barr, Azlisham Mohd Nor, Claire Brown, Nicola Persad, Charlotte Eglinton, Marie Weinling, Benjamin Hyams, Alex Shah, John Baker, Anthony Byrne, Caroline McGhee, Amanda Smart, Claire Copeland, Michael Carpenter, Marion Walker, Richard Davey, Ann Needle, Razik Fathima, Gavin Bateman, Prabal Datta, Andrew Stanners, Linda Jackson, Julie Ball, Michelle Davis, Natalie Atkinson, Michelle Fawcett, Teresa Thompson, Helen Guy, Valerie Hogg, Carole Hays, Stephen Woodward, Mohammad Haque, Eluzai Hakim, Stuart Symonds, Mehran Maanoosi, Jane Herman, Toby Black, Skelton Miriam, Caroline Clarke, Alpha Anthony, Michele Tribbeck, Julie Cronin, Denise Mead, Ruth Fennelly, James McIlmoyle, Christina Dickinson, Carol Jeffs, Sajjad Anwar, Joanne Howard, Kirsty Jones, Saikat Dhar, Caroline Clay, Muhammad Siddiq, Simone Ivatts, Yolanda Baird, Moore Sally, Isobel Amey, Sophie Newton, Lisa Clayton-Evans, Indra Chadbourn, Rayessa Rayessa, Charde Naylor, Alicia Rodgers, Lisa Wilson, Sarah Wilson, Emma Clarkson, Ruth Davies, Paula Owings, Graeme Sangster, Valerie Gott, Victoria Little, Pauline Weir, Suja Cherian, Deepa Jose, Helen Moroney, Susan Downham, Angela Dodd, Venetia Vettimootal Johnson, Laura Codd, Naomi Robinson, Ashraf Ahmed, Mo Albazzaz, Sharon Johnson, Carol Denniss, Mishell Cunningham, Tajammal Zahoor, Timothy Webster, Sandra Leason, Syed Haider, Kausic Chatterjee, Arumugam Nallasivan, Charlotte Perkins, Samantha Seagrave, Colin Jenkins, Fiona Price, Claire Hughes, Lily Mercer, Malik Hussain, Sarah Brown, Miriam Harvey, Jane Homan, Mohammad Khan, Robert Whiting, Leanne Foote, Nicholas Hunt, Helen Durman, Lucy Brotherton, Jayne Foot, Corinne Pawley, Eliza Foster, Alison Whitcher, Kneale Metcalf, Jenny Jagger, Susan McDonald, Kelly Waterfield, Patrick Sutton, Naval Shinh, Ajmal Anversha, Garth Ravenhill, Richard Greenwood, Janak Saada, Alison Wiltshire, Rebekah Perfitt, Sreeman Andole, Naveen Gadapa, Karen Dunne, Magdalini Krommyda, Evelyne Burssens, Sam King, Catherine Plewa, Nigel Smyth, Jenny Wilson, Elio Giallombardo, Charlotte Eglinton, Lucy Sykes, Pradeep Kumar, James Barker, Isabel Huggett, Linda Dunn, Charlotte Culmsee, Philip Thomas, Min Myint, Richard O'Brien, Helen Brew, Nikhil Majmudar, Janice OConnell, George Bunea, Charlotte Fox, Diane Gulliver, Andrew Smith, Betty Mokoena, Naweed Sattar, Ramesh Krishnamurthy, Emily Osborne, David Wilson, Belinda Wroath, Kevin Dynan, Michael Power, Susan Thompson, Victoria Adell, Enoch Orugun, Una Poultney, Rachel Glover, Hannah Crowther, Sarah Thornthwaite, Ivan Wiggam, Aine Wallace, Enda Kerr, Ailsa Fulton, Annemarie Hunter, Suzanne Tauro, Sarah Cuddy, David Mangion, Anne Hardwick, Skarlet Markova, Tara Lawrence, Carmen Constantin, Jo Fletcher, Isobel Thomas, Kerry Pettitt, Lakshmanan Sekaran, Margaret Tate, Kiranjit Bharaj, Rohan Simon, Frances Justin, Sakthivel Sethuraman, Duke Phiri, Niaz Mohammed, Meena Chauhan, Khaled Elfandi, Uzma Khan, Samantha Stafford, Julie Reddan, David Eveson, Amit Mistri, Lisa Manning, Shagufta Khan, Champa Patel, Mohammed Moqsith, Saira Sattar, Man Yee Lam, Kashif Musarrat, Claire Stephens, Latheef Kalathil, Richard Miller, Maqsud Salehin, Nikki Gautam, Duncan Bailey, Kelly Amor, Julie Meir, Anne Nicolson, Javed Imam, Lisa Wood, Julie White, Mahmud Sajid, George Ghaly, Margaret Ball, Rachel Gascoyne, Harald Proeschel, Simon Sharpe, Sarah Horton, Emily Beaves, Stephanie Jones, Brigitte Yip, Murdina Bell, Linda MacLiver, Brian MacInnes, Derek Esson, Don Sims, Jennifer Hurley, Mark Willmot, Claire Sutton, Edward Littleton, Susan Maiden, Rachael Jones, James Cunningham, Carole Green, Michelle Bates, Raj Shekhar, Kelly Waterfield, Ellie Gilham, Iman Ahmed, Rachel Crown, Tracy Fuller, Neetish Goorah, Angela Bell, Christine Kelly, Arun Singh, Jamie Walford, Benjamin Tomlinson, Farzana Patel, Stephen Duberley, Ingrid Kane, Chakravarthi Rajkumar, Jane Gaylard, Joanna Breeds, Nicola Gainsborough, Alexandra Pitt-Ford, Emma Barbon, Laura Latter, Philip Thompson, Simon Hervey, Shrivakumar Krishnamoorthy, Joseph Vassallo, Deborah Walter, Helen Cochrane, Meena Srinivasan, Robert Campbell, Denise Donaldson, Nichola Motherwell, Frances Hurford, Indranil Mukherjee, Antony Kenton, Sheila Nyabadza, Irene Martin, Benjamin Hunt, Hardi Hassan, Sarah O'Toole, Bander Dallol, Janet Putterill, Ratneshwari Jha, Rachel Gallifent, Puneet Kakar, Aparna Pusalkar, kelly Chan, Puneet Dangri, Hannah Beadle, Angela Cook, Karen Crabtree, Santhosh Subramonian, Peter Owusu-Agyei, Natalie Temple, Nicola Butterworth-Cowin, Suzanne Ragab, Kerstin Knops, Emma Jinks, Christine Dickson, Laura Gleave, Judith Dube, Jacqui Leggett, Tatiana Garcia, Sissy Ispoglou, Rachel Evans, Sandeep Ankolekar, Anne Hayes, Hlaing Ni, Bithi Rahman, Josette Milligan, Carol Graham, Josin Jose, Breffni Keegan, Mandy Doherty, Jim Kelly, Caroline Blair, Richard Dewar, James White, Kelly Thomas

**Affiliations:** a Centre for Clinical Brain Sciences, University of Edinburgh, Edinburgh, UK; b Usher Institute of Population Health Sciences and Informatics, University of Edinburgh, Edinburgh, UK; c Centre for Cardiovascular Science, University of Edinburgh, Edinburgh, UK; d UK Dementia Research Institute at the University of Edinburgh, University of Edinburgh, Edinburgh, UK; e Department of Neuroradiology, Newcastle-upon-Tyne Hospitals NHS Trust, Newcastle-upon-Tyne, UK; f Institute of Neuroscience and Newcastle University Institute for Ageing, Newcastle University, Newcastle-upon-Tyne, UK; g Department of Clinical Neurosciences, NHS Lothian, Edinburgh, UK; h Department of Radiology, Addenbrooke's Hospital, Cambridge, UK; i Division of Clinical Neurosciences, Faculty of Medicine and Health Sciences, University of Nottingham, Nottingham, UK; j Stroke Research Group, Department of Brain Repair and Rehabilitation, University College London Queen Square Institute of Neurology, London, UK

## Abstract

**Background:**

Findings from the RESTART trial suggest that starting antiplatelet therapy might reduce the risk of recurrent symptomatic intracerebral haemorrhage compared with avoiding antiplatelet therapy. Brain imaging features of intracerebral haemorrhage and cerebral small vessel diseases (such as cerebral microbleeds) are associated with greater risks of recurrent intracerebral haemorrhage. We did subgroup analyses of the RESTART trial to explore whether these brain imaging features modify the effects of antiplatelet therapy.

**Methods:**

RESTART was a prospective, randomised, open-label, blinded-endpoint, parallel-group trial at 122 hospitals in the UK that assessed whether starting antiplatelet therapy might reduce the risk of recurrent symptomatic intracerebral haemorrhage compared with avoiding antiplatelet therapy. For this prespecified subgroup analysis, consultant neuroradiologists masked to treatment allocation reviewed brain CT or MRI scans performed before randomisation to confirm participant eligibility and rate features of the intracerebral haemorrhage and surrounding brain. We followed participants for primary (recurrent symptomatic intracerebral haemorrhage) and secondary (ischaemic stroke) outcomes for up to 5 years (reported elsewhere). For this report, we analysed eligible participants with intracerebral haemorrhage according to their treatment allocation in primary subgroup analyses of cerebral microbleeds on MRI and in exploratory subgroup analyses of other features on CT or MRI. The trial is registered with the ISRCTN registry, number ISRCTN71907627.

**Findings:**

Between May 22, 2013, and May 31, 2018, 537 participants were enrolled, of whom 525 (98%) had intracerebral haemorrhage: 507 (97%) were diagnosed on CT (252 assigned to start antiplatelet therapy and 255 assigned to avoid antiplatelet therapy, of whom one withdrew and was not analysed) and 254 (48%) underwent the required brain MRI protocol (122 in the start antiplatelet therapy group and 132 in the avoid antiplatelet therapy group). There were no clinically or statistically significant hazards of antiplatelet therapy on recurrent intracerebral haemorrhage in primary subgroup analyses of cerebral microbleed presence (2 or more) versus absence (0 or 1) (adjusted hazard ratio [HR] 0·30 [95% CI 0·08–1·13] *vs* 0·77 [0·13–4·61]; p_interaction_=0·41), cerebral microbleed number 0–1 versus 2–4 versus 5 or more (HR 0·77 [0·13–4·62] *vs* 0·32 [0·03–3·66] *vs* 0·33 [0·07–1·60]; p_interaction_=0·75), or cerebral microbleed strictly lobar versus other location (HR 0·52 [0·004–6·79] *vs* 0·37 [0·09–1·28]; p_interaction_=0·85). There was no evidence of heterogeneity in the effects of antiplatelet therapy in any exploratory subgroup analyses (all p_interaction_>0·05).

**Interpretation:**

Our findings exclude all but a very modest harmful effect of antiplatelet therapy on recurrent intracerebral haemorrhage in the presence of cerebral microbleeds. Further randomised trials are needed to replicate these findings and investigate them with greater precision.

**Funding:**

British Heart Foundation.

## Introduction

Cerebral small vessel diseases cause, or contribute to, the majority of stroke due to intracerebral haemorrhage. Combinations of the many imaging biomarkers on brain CT and MRI can identify cerebral small vessel diseases such as cerebral amyloid angiopathy with reasonable accuracy.^1^, ^2^, ^3^

Some brain imaging features of intracerebral haemorrhage and cerebral small vessel diseases are associated with a higher risk of intracerebral haemorrhage recurrence in general, and in people taking antiplatelet therapy.^4^, ^5^, ^6^, ^7^, ^8^ For example, the proportional increase in the risk of intracerebral haemorrhage recurrence is up to five-times higher after lobar versus non-lobar haemorrhage,^8^ up to six-times higher with presence versus absence of cerebral microbleeds on MRI,^7^ and roughly four-times higher with presence versus absence of superficial siderosis on MRI.^6^ Consequently, guidelines and opinions suggest that location of intracerebral haemorrhage and some features of cerebral small vessel diseases can guide therapeutic decisions.^9^, ^10^, ^11^, ^12^ However, decisions about antiplatelet therapy for patients with intracerebral haemorrhage and cerebral microbleeds are mainly informed by two analyses of a single-centre small observational cohort study of survivors of intracerebral haemorrhage,^5^, ^13^ one of which found an up to five-times greater risk of recurrent lobar intracerebral haemorrhage associated with use of aspirin in people with cerebral microbleeds.^5^

**Research in context**

**Evidence before this study**

Brain imaging features of intracerebral haemorrhage (such as lobar location) and brain imaging biomarkers of cerebral small vessel diseases (such as microbleeds or superficial siderosis) are associated with a higher risk of intracerebral haemorrhage recurrence. Consequently, some physicians withhold antiplatelet therapy from people with these imaging features. However, it is unclear whether the effects of antiplatelet therapy vary by these brain imaging features. We searched MEDLINE Ovid (from 1948), Embase Ovid (from 1980), and bibliographies of relevant publications on Feb 28, 2019, combining search terms for cerebral small vessel diseases, intracerebral haemorrhage, randomised controlled trials, antiplatelet therapy, and brain imaging in humans (appendix). There were no language restrictions. The SPS3 randomised controlled trial showed no heterogeneity by brain MRI features in the effects of aspirin and clopidogrel versus aspirin alone in 1278 adults after subcortical ischaemic stroke. In the IST and CAST randomised controlled trials, there was no heterogeneity by visible infarction on brain CT in the effects of aspirin versus control in 31 072 adults after acute ischaemic stroke. However, no published randomised trials have reported the effects of antiplatelet therapy after intracerebral haemorrhage by brain imaging features.

**Added value of this study**

To our knowledge, RESTART is the first randomised controlled trial to investigate the effects of starting versus avoiding antiplatelet therapy in adults with previous intracerebral haemorrhage that occurred while taking antithrombotic (antiplatelet or anticoagulant) therapy, grouped by their brain imaging features. We did not find clinically or statistically significant hazardous effects of antiplatelet therapy on recurrent intracerebral haemorrhage or ischaemic stroke in primary subgroup analyses of cerebral microbleed presence, nor in any exploratory subgroup analyses of intracerebral haemorrhage location, previous vascular lesions, atrophy, periventricular lucencies, white matter hyperintensities, superficial siderosis, or diagnostic criteria for cerebral amyloid angiopathy.

**Implications of all the available evidence**

Our results exclude all but a very modest harmful effect of antiplatelet therapy on the primary outcome of recurrent intracerebral haemorrhage in the presence of cerebral microbleeds. Our findings provide information about the safety of antiplatelet therapy in subgroups of adults with intracerebral haemorrhage, although the precision of these analyses was limited by small sample size. The directions of the effects we have found permit the inclusion of adults with a wide range of brain imaging features in ongoing trials (RESTART-Fr, NCT02966119; and STATICH, NCT03186729) and future randomised controlled trials of antiplatelet therapy after intracerebral haemorrhage, which are likely to require sample sizes of more than 2200 participants to detect statistically significant interactions with treatment effects.

A randomised controlled trial is the most reliable test of overall treatment effects and enables estimation of heterogeneity in the effects of treatment in primary subgroup analyses (informed by previous evidence), as well as in other exploratory subgroup analyses.^14^ One randomised trial found no heterogeneity by MRI features in the effects of aspirin and clopidogrel versus aspirin alone on stroke recurrence in 1278 adults after subcortical ischaemic stroke,^15^ and two other trials found no heterogeneity by the presence of visible infarction on CT in the effects of aspirin versus control on stroke recurrence in 31 072 adults after acute ischaemic stroke.^16^, ^17^ Randomised trials have not been done—but are needed—to investigate the effects of antiplatelet therapy after intracerebral haemorrhage according to brain imaging features of intracerebral haemorrhage or cerebral small vessel diseases.^18^

In the REstart or STop Antithrombotics Randomised Trial (RESTART), survivors of intracerebral haemorrhage that occurred while taking antithrombotic therapy who were randomly allocated to start antiplatelet therapy had fewer recurrences of symptomatic intracerebral haemorrhage over a median follow-up of 2·0 years than did those allocated to avoid antiplatelet therapy (12 [4%] of 268 *vs* 23 [9%] of 268, respectively; adjusted hazard ratio [HR] 0·51, 95% CI 0·25–1·03; p=0·060).^19^ In a prespecified primary subgroup analysis, there was no evidence of heterogeneity of the effects of antiplatelet therapy on the primary outcome by investigators' categorisation of lobar versus non-lobar intracerebral haemorrhage location (p_interaction_=0·23).^19^ To further explore effects according to features of the intracerebral haemorrhage and cerebral small vessel diseases, we collected brain imaging performed on all participants before randomisation, and did primary subgroup analyses of cerebral microbleeds on MRI and exploratory subgroup analyses of other brain imaging features.

## Methods

### Study design and participants

RESTART was an investigator-led, pragmatic, multicentre, prospective, randomised, open-label, blinded-endpoint, parallel-group trial in 122 hospitals in the UK. Participant eligibility, consent, data collection, monitoring, approvals, procedures, and statistical analysis principles are described in detail in the protocol,^20^ statistical analysis plan,^21^ and primary report of the trial.^19^

Briefly, patients were eligible for enrolment if they were aged 18 years or older, had survived at least 24 h after spontaneous intracerebral haemorrhage, and were taking antithrombotic (antiplatelet or anticoagulant) therapy for the prevention of occlusive vascular disease at the onset of intracerebral haemorrhage, after which therapy was discontinued. Patients were ineligible if the intracerebral haemorrhage was attributable to preceding head injury, haemorrhagic transformation of an ischaemic stroke, or intracranial haemorrhage without intracerebral haemorrhage; or if they were still taking antithrombotic therapy at the time of consent (ie, after intracerebral haemorrhage). Patients, or a representative, provided written informed consent in inpatient or outpatient hospital settings. The Scotland A Research Ethics Committee approved the trial protocol.

Before randomisation, collaborators had to confirm that the brain imaging (usually CT, but sometimes MRI alone) that diagnosed the qualifying intracerebral haemorrhage was available and would be sent to the trial coordinating centre. Participants who had not already undergone brain MRI that complied with the trial's imaging protocol, and who were able and willing to undergo brain MRI, provided informed consent for this to be performed. Details of the randomisation method and masking are described in the protocol and primary report of the trial.^19^, ^20^

### Procedures

The intervention of starting antiplatelet therapy was restricted to the use of one or more of oral aspirin, dipyridamole, or clopidogrel, begun within 24 h of randomisation. The comparator was a policy of avoiding antiplatelet therapy (ie, no placebo group).^19^, ^20^

To be permitted to enrol participants in the MRI substudy, sites had to provide test imaging that passed image acquisition standards and adhered to the minimum requirements for sequences and parameters specified in the imaging protocol. Any field strength was permitted. Coverage from the very top of the vertex to the foramen magnum was essential. An axial gradient-recalled echo (GRE) T2* sequence was required with specified slice thickness (3 mm optimal, 3–5 mm acceptable), slice gap (none optimal, not more than 1 mm acceptable), and echo time (20–30 ms optimal, 15–40 ms acceptable). The following MRI sequences were essential (although their sequence parameters were not specified): T1-weighted (volumetric preferred, otherwise sagittal), axial T2-weighted, axial diffusion-weighted imaging, and fluid-attenuated inversion recovery (axial preferred). Brain MRI was only included if acquired from participants before randomisation to avoid the possibility that the allocated treatment might affect appearances.

Investigators copied the earliest imaging study that diagnosed the qualifying intracerebral haemorrhage and any brain MRI substudy images that were obtained before randomisation in Digital Imaging and Communications in Medicine (DICOM) format, removed personal identifiers and replaced these with a participant's study number (pseudo-anonymisation), and sent the images to the trial coordinating centre after randomisation.

The RESTART imaging manager checked each imaging study to ensure that the participant, timing, and modality corresponded to the information provided before randomisation. Each brain MRI study was checked to ensure that all the required sequences had been provided, using acceptable parameters. After quality assurance, all CT and MRI studies were uploaded to an electronic archive and allocated to one of a panel of consultant neuroradiologists via the in-house, web-based, systematic image review system for confirmation and characterisation of brain imaging features of intracerebral haemorrhage diagnosis and cerebral small vessel diseases.^1^, ^22^

A member of the independent panel of consultant neuroradiologist adjudicators reviewed all imaging masked to treatment allocation before the trial database was locked and the randomisation code was broken.^20^ The panel member checked eligibility of each participant by confirming that each imaging study demonstrated parenchymal haemorrhage, consistent with the given date of symptom onset, most likely due to intracerebral haemorrhage and not due to haemorrhagic transformation of ischaemic stroke. If the independent rating of the imaging studies did not confirm the existence of intracerebral haemorrhage, another member of the panel re-reviewed all relevant imaging studies and made a final determination of eligibility.

For CT imaging, the neuroradiologist used validated scales to rate features of the single (or largest, if multiple) intracerebral haemorrhage (side, location, volume in mL measured by the ABC/2 method,^23^ intraventricular extension, subarachnoid extension, and subdural extension), the surrounding brain (previous vascular lesions^22^, ^24^ and periventricular lucencies [leukoaraiosis]^25^), and atrophy.^22^, ^26^ One neuroradiologist rated the features of acute intracerebral haemorrhages with a lobar epicentre (subarachnoid extension and finger-like projections) to estimate the probability of underlying cerebral amyloid angiopathy according to the CT-only version of the Edinburgh diagnostic criteria.^2^

For MRI, the neuroradiologist used validated scales to rate features of the single (or largest, if multiple) intracerebral haemorrhage (side, location, volume in mL measured by the ABC/2 method,^23^ intraventricular extension, subarachnoid extension, and subdural extension), the surrounding brain (previous vascular lesions—ie, previous infarcts or previous haemorrhages that were not microbleeds),^1^, ^27^ superficial siderosis (focal or disseminated),^3^ white matter hyperintensities of presumed vascular origin,^28^ basal ganglia mineral deposits, enlarged perivascular spaces, atrophy,^29^ and cerebral microbleed presence, number, and location,^30^, ^31^ as defined previously.^1^

### Outcomes

The RESTART trial's primary outcome (fatal or non-fatal radiographically or pathologically proven recurrent symptomatic intracerebral haemorrhage) and secondary outcomes have been reported elsewhere.^19^ For this report, we analysed eligible participants with intracerebral haemorrhage according to their treatment allocation in primary subgroup analyses of presence, burden, and location of cerebral microbleeds on MRI and in exploratory subgroup analyses of other brain imaging features on CT or MRI. The secondary outcome in the subgroup analyses of brain imaging features was ischaemic stroke. Outcomes were ascertained and adjudicated as described in the protocol and primary report of the trial.^19^, ^20^

### Statistical analysis

In our protocol and statistical analysis plan,^20^, ^21^ we prespecified that the MRI substudy would focus on primary subgroup analyses, testing whether there was heterogeneity in the effects of antiplatelet therapy on the trial's primary outcome of recurrent intracerebral haemorrhage by the presence, number, or location of cerebral microbleeds. We collected other brain imaging features on MRI and CT for exploratory subgroup analyses of the effects of antiplatelet therapy on recurrent intracerebral haemorrhage or ischaemic stroke. We present the analyses of CT imaging features first because of their larger sample size, but focus our reporting on the primary subgroup analyses of cerebral microbleeds on brain MRI.

We intended to obtain diagnostic imaging studies for all participants and recruit approximately 75% of RESTART participants to the MRI substudy, although ultimately investigators recruited a smaller total number and proportion of all participants, diminishing the precision of our findings. In RESTART, brain imaging was not always done despite consent being obtained; was not always provided; was performed but might have contravened the required protocol; was performed, but might have been degraded by motion artefact; or was performed but demonstrated that the patient was ineligible for inclusion in RESTART (which precluded collection of ratings by the RESTART imaging panel). We quantified these exclusions, retaining participants in the imaging analyses if pre-randomisation brain imaging was obtained (and was compliant with the RESTART protocol in the case of MRI), was readable, and confirmed intracerebral haemorrhage. We recorded the timing of imaging (symptom onset to earliest imaging study and earliest imaging study to randomisation).

We focused descriptive analyses on imaging features of primary interest at a meeting between RA-SS, PMW, and JMW before database lock and unmasking the trial database. We chose not to analyse other features at this time (basal ganglia mineral deposits and enlarged perivascular spaces on MRI). We also agreed on pragmatic categorisations of some complex variables (eg, previous vascular lesions, periventricular lucencies, and atrophy) based on previous experience of simplifying the complex rating scales of these features for analysis.^1^, ^2^, ^17^, ^22^, ^24^, ^26^, ^29^

We prespecified that cerebral microbleed presence was two or more microbleeds (in view of inter-rater variation in the reporting of solitary microbleeds^30^, ^31^) and that microbleed location would be grouped as strictly lobar versus other, for dichotomous analysis of the presence of cerebral microbleeds on MRI. We prespecified that for categorical analysis of cerebral microbleed number, the split would be 0 or 1 versus 2–4 versus 5 or more.^21^ We investigated whether cerebral microbleed presence and burden (as a continuous variable) were associated, as expected,^7^, ^32^ with the first recurrent intracerebral haemorrhage or ischaemic stroke in Cox proportional hazards regression models adjusted for the five covariates in the minimisation algorithm (qualifying intracerebral haemorrhage location, time since symptom onset, antiplatelet therapy preferred by the participant's physician if allocated to start, participant age at randomisation, and predicted probability of being alive and independent at 6 months). We analysed heterogeneity of the effects of antiplatelet therapy on the first recurrent intracerebral haemorrhage between subgroups using a statistical test of interaction, by including an interaction term between treatment group and each imaging feature in Cox proportional hazards regression models adjusted for the five covariates in the minimisation algorithm. We applied the Firth correction to Cox proportional hazards models in which we observed monotone likelihoods and calculated HRs with 95% profile likelihood confidence limits.^33^ The unmasked trial statistician performed statistical analyses with SAS, version 9.4.

The trial is registered with the ISRCTN registry, number ISRCTN71907627.

### Role of the funding source

The funder of the study had no role in study design, data collection, data analysis, data interpretation, or writing of the report. The corresponding author had full access to all data in the study and had final responsibility for the decision to submit for publication.

## Results

**Figure 1:**
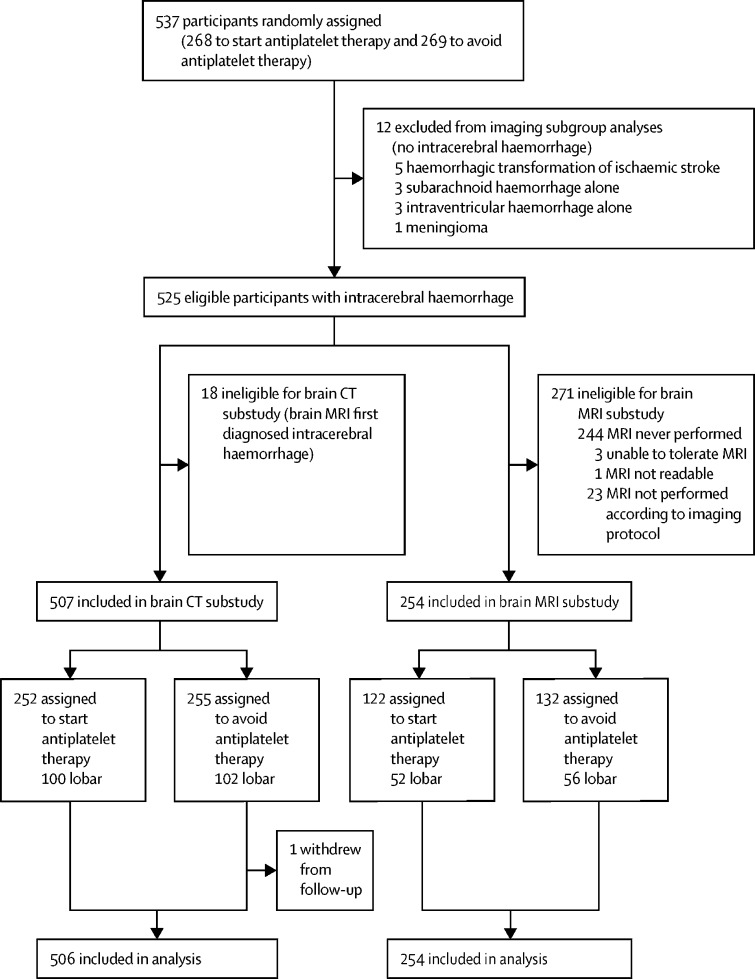
Profile of imaging substudies within RESTART

Between May 22, 2013, and May 31, 2018, 537 participants were enrolled in the RESTART trial and randomly assigned to start antiplatelet therapy (n=268) or to avoid antiplatelet therapy (n=269), of whom 12 were ineligible for the imaging subgroup analyses because their intracranial haemorrhage did not extend into the brain parenchyma (figure 1). 18 participants were diagnosed using MRI alone, leaving 507 in the brain CT substudy. 271 participants did not undergo per-protocol MRI, leaving 254 in the brain MRI substudy.

In the brain CT substudy, the median time from symptom onset to earliest CT was 0 days (IQR 0–1) and the median time from earliest CT to randomisation was 74 days (27–144). The age and sex distributions of participants included in the CT substudy were similar to those of participants in the whole trial (table 1).^19^ Most participants had a solitary intracerebral haemorrhage, 40% of which were lobar. After excluding eight participants whose first brain CT showed subacute intracerebral haemorrhage (which precluded accurate rating of brain imaging features of the haemorrhage^2^), 305 had non-lobar haemorrhage and the remaining 194 participants with CT of acute intracerebral haemorrhage with a lobar epicentre were rated for the probability of underlying cerebral amyloid angiopathy according to the Edinburgh diagnostic criteria:^2^ 29 (6%) of 499 participants had a high probability (finger-like projections and subarachnoid extension) and 165 (33%) had a lower probability. Participants' brains commonly showed previous vascular lesions (316 [62%] of 507), severe periventricular lucencies (195 [38%]), and moderate-to-severe atrophy (80 [16%]). There were small baseline imbalances in intraventricular extension, subarachnoid extension, and periventricular lucencies (table 1). One participant in the avoid antiplatelet therapy group of the brain CT substudy withdrew from follow-up and was not included in the analyses.

**Table 1:** Baseline characteristics of participants in the CT substudy

		**Start antiplatelet therapy (n=252)**	**Avoid antiplatelet therapy (n=255)**
**Participant characteristics**			
Sex			
	Female	89 (35%)	79 (31%)
	Male	163 (65%)	176 (69%)
Age (years)		77 (69–83)	76 (70–82)
Number of intracerebral haemorrhages			
One		235 (93%)	242 (95%)
More than one		17 (7%)	13 (5%)
**Characteristics of the largest intracerebral haemorrhage**			
Side			
	Left	120 (48%)	117 (46%)
	Right	132 (52%)	138 (54%)
Location			
	Deep	123 (49%)	123 (48%)
	Infratentorial	29 (12%)	30 (12%)
	Lobar	100 (40%)	102 (40%)
Volume of largest intracerebral haemorrhage (mL)		3·7 (1·1–10·8)	4·3 (1·2–11·6)
Intraventricular extension		55 (22%)	70 (27%)
Subarachnoid extension		42 (17%)	50 (20%)
Subdural extension		6 (2%)	8 (3%)
Edinburgh CT-only criteria^2^ for acute intracerebral haemorrhages with lobar epicentres (n=499)*			
	Non-lobar intracerebral haemorrhage	152 (61%)	153 (61%)
	Lower probability of cerebral amyloid angiopathy	83 (33%)	82 (33%)
	High probability of cerebral amyloid angiopathy	14 (6%)	15 (6%)
**Characteristics of the brain**			
Previous vascular lesions			
	No	98 (39%)	93 (36%)
	Yes	154 (61%)	162 (64%)
Periventricular lucencies score†			
	0–2	165 (65%)	147 (58%)
	3–4	87 (35%)	108 (42%)
Atrophy score‡			
	0–2	215 (85%)	212 (83%)
	3–4	37 (15%)	43 (17%)

Data are n (%) or median (IQR).

* Eight participants whose first brain CT showed subacute intracerebral haemorrhage (which precluded accurate rating of brain imaging features of the haemorrhage) were excluded. Start antiplatelet therapy, n=249; avoid antiplatelet therapy, n=250. High probability of cerebral amyloid angiopathy is defined as finger-like projections and subarachnoid extension; lower probability is all other features.

† Periventricular lucencies score combines both anterior and posterior white matter scores (0=no lucency; 1=lucency restricted to region adjoining ventricles; 2=lucency covering entire region from lateral ventricle to cortex).

‡ Atrophy score combines both central and cortical atrophy (each scored 0=none; 1=moderate; 2=severe).

**Figure 2:**
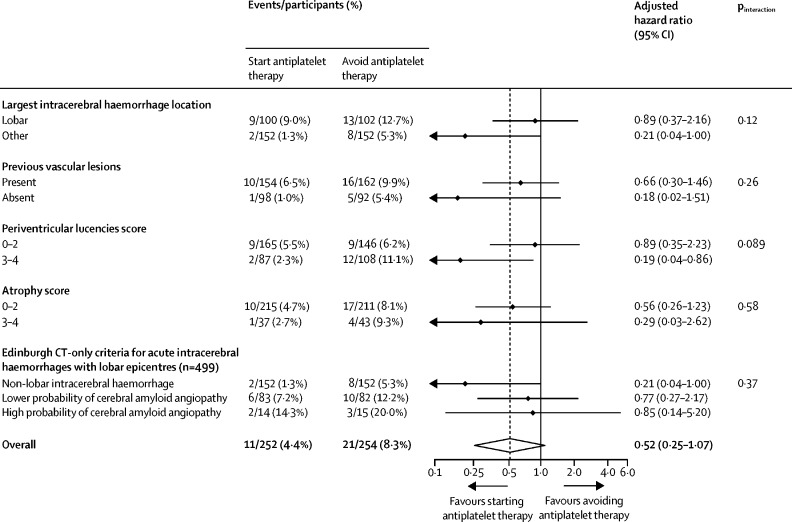
Prespecified exploratory subgroup analyses of the risk of first recurrent symptomatic intracerebral haemorrhage (the primary outcome) by brain CT features

In prespecified exploratory subgroup analyses of CT features, we did not find strong evidence of statistically significant heterogeneity in the effects of antiplatelet therapy on recurrent intracerebral haemorrhage (figure 2) or ischaemic stroke (appendix) by intracerebral haemorrhage location, previous vascular lesions, periventricular lucencies, atrophy, or the probability of underlying cerebral amyloid angiopathy.

In the brain MRI substudy, the median time from symptom onset to MRI was 55 days (IQR 18–102) and the median time from MRI to randomisation was 2 days (0–18). The age and sex distributions of participants included in the MRI substudy were similar to those of participants in the whole trial (table 2).^19^ 108 (43%) of 254 substudy participants had lobar intracerebral haemorrhage and 235 (93%) had a GRE T2* MRI sequence of sufficient quality to rate cerebral microbleeds: 93 (40%) of 235 had two or more microbleeds, 62 (26%) of 235 had five or more microbleeds, and 20 (22%) of 93 had strictly lobar microbleeds, such that 47 (20%) of 235 probably had cerebral amyloid angiopathy and 30 (13%) of 235 possibly had cerebral amyloid angiopathy according to the modified Boston criteria.^3^ Background brain characteristics on MRI (table 2) were very similar to characteristics on CT (table 1). There were small baseline imbalances in sex, intracerebral haemorrhage location and extension, atrophy, and cerebral microbleed strictly lobar location (table 2).

**Table 2:** Baseline characteristics of participants in the MRI substudy

			**Start antiplatelet therapy (n=122)**	**Avoid antiplatelet therapy (n=132)**
**Participant characteristics**				
Sex				
	Female		42 (34%)	36 (27%)
	Male		80 (66%)	96 (73%)
Age (years)			76 (70–81)	75 (69–82)
Number of intracerebral haemorrhages				
	One		117 (96%)	124 (94%)
	More than one		5 (4%)	8 (6%)
**Characteristics of the largest intracerebral haemorrhage**				
Side				
	Left		56 (46%)	59 (45%)
	Right		66 (54%)	73 (55%)
Location				
	Deep		53 (43%)	66 (50%)
	Infratentorial		17 (14%)	10 (8%)
	Lobar		52 (43%)	56 (42%)
Volume of largest intracerebral haemorrhage (mL)			2·3 (0·4–7·9)	1·6 (0·4–7·1)
Intraventricular extension			10 (8%)	13 (10%)
Subarachnoid extension			17 (14%)	26 (20%)
Subdural extension			2 (2%)	8 (6%)
**Characteristics of the brain**				
Previous ischaemic lesions				
None			72 (59%)	74 (56%)
One			18 (15%)	25 (19%)
More than one			32 (26%)	33 (25%)
Previous haemorrhagic lesions (that are not cerebral microbleeds)				
None			110 (90%)	112 (85%)
One			12 (10%)	18 (14%)
More than one			0	2 (2%)
Superficial siderosis				
None			95 (78%)	99 (75%)
Focal			19 (16%)	23 (17%)
Disseminated			8 (7%)	10 (8%)
White matter hyperintensities score*				
0–2			39 (32%)	43 (33%)
3–6			83 (68%)	89 (67%)
Atrophy score†				
0–2			76 (62%)	71 (54%)
3–4			46 (38%)	61 (46%)
Cerebral microbleeds (n=235)‡				
	Presence			
		0–1	66 (58%)	76 (63%)
		2–4	16 (14%)	15 (12%)
		5 or more	32 (28%)	30 (25%)
	Location§			
		Strictly lobar	7 (15%)	13 (29%)
		Other	41 (85%)	32 (71%)
	Modified Boston criteria^3^ for participants with ratings for microbleeds and superficial siderosis			
		Probable cerebral amyloid angiopathy	19 (17%)	28 (23%)
		Possible cerebral amyloid angiopathy	14 (12%)	16 (13%)
		Neither probable nor possible cerebral amyloid angiopathy	81 (71%)	77 (64%)
(Table 2 continues in next column)				

Data are n (%) or median (IQR).

* White matter hyperintensities score combines periventricular and deep (subcortical) white matter (each scored as 0, 1, 2, or 3).

† Atrophy score combines central and cortical (each scored 0=none; 1=moderate; 2=severe).

‡ 235 participants had an MRI sequence of sufficient quality to rate cerebral microbleeds; start antiplatelet therapy, n=114; avoid antiplatelet therapy, n=121.

§ Denominators are start antiplatelet therapy, n=48; avoid antiplatelet therapy, n=45.

As was expected in this population,^7^, ^32^ in the primary subgroup of 235 participants with cerebral microbleeds, their presence (2 or more versus 0 or 1) and burden (linear trend of 0 or 1, 2–4, and 5 or more) were associated with first recurrent intracerebral haemorrhage (adjusted HR 3·62 [95% CI 1·34–9·79] and 1·99 [1·20–3·31], respectively) and ischaemic stroke (HR 1·92 [0·83–4·46] and 1·62 [1·03–2·55], respectively; appendix).

**Figure 3:**
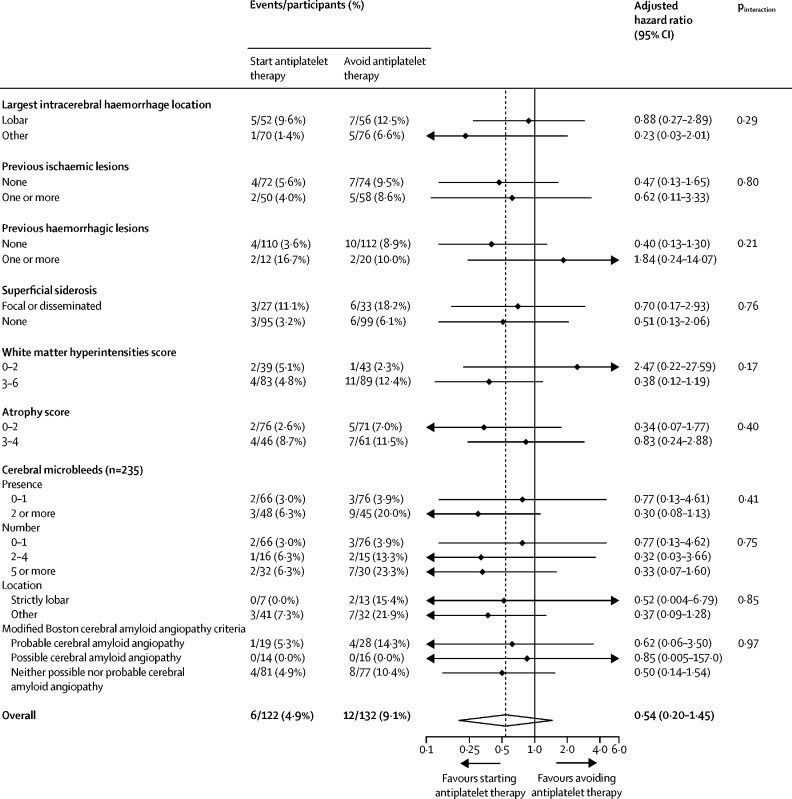
Prespecified primary and exploratory subgroup analyses of the risk of first recurrent symptomatic intracerebral haemorrhage (the primary outcome) by brain MRI features

We did not find clinically or statistically significant hazardous effects of antiplatelet therapy on recurrent intracerebral haemorrhage in any primary subgroup analyses of cerebral microbleed presence versus absence (HR 0·30 [95% CI 0·08–1·13] *vs* 0·77 [0·13–4·61]; p_interaction_=0·41), cerebral microbleed number 0–1 versus 2–4 versus 5 or more (HR 0·77 [0·13–4·62] *vs* 0·32 [0·03–3·66] *vs* 0·33 [0·07–1·60]; p_interaction_=0·75), or strictly lobar versus other location (HR 0·52 [0·004–6·79] *vs* 0·37 [0·09–1·28]; p_interaction_=0·85; figure 3).

In prespecified exploratory subgroup analyses of other MRI features, we did not find strong evidence of statistically significant heterogeneity in the effects of antiplatelet therapy on recurrent intracerebral haemorrhage (figure 3) or ischaemic stroke (appendix; all p_interaction_>0·05).

## Discussion

In these subgroup analyses of the RESTART trial, the estimated effect of antiplatelet therapy in the subgroup with cerebral microbleeds (HR 0·30, 95% CI 0·08–1·13) excluded all but a very modest harmful effect of antiplatelet therapy on the primary outcome of recurrent intracerebral haemorrhage. Moreover, we did not find strong evidence of any significant heterogeneity of the effects of antiplatelet therapy on recurrent intracerebral haemorrhage or ischaemic stroke in exploratory subgroup analyses of other CT or MRI features of the intracerebral haemorrhage or cerebral small vessel diseases.

Although caution is needed in the interpretation of non-significant differences between small subgroups,^14^ we did not find strong evidence within the primary subgroup analyses of cerebral microbleeds that was consistent with the five-times greater risk of recurrent lobar intracerebral haemorrhage associated with aspirin use in people with cerebral microbleeds as seen in an observational study,^5^ which has hitherto influenced clinical practice.^9^, ^34^ Furthermore, we did not find strong evidence of differences within exploratory subgroup analyses to suggest that superficial siderosis or diagnostic criteria for cerebral amyloid angiopathy might modify the risk of intracerebral haemorrhage with antiplatelet therapy.^5^, ^35^ Although these brain imaging features are associated with higher absolute risks of intracerebral haemorrhage recurrence in observational studies,^6^, ^7^ we did not find strong evidence that there was heterogeneity in the effects of antiplatelet therapy in these subgroups.

To our knowledge, RESTART is the first randomised trial comparing starting versus avoiding antiplatelet therapy after intracerebral haemorrhage to explore whether the effects of antiplatelet therapy vary by imaging features of intracerebral haemorrhage or cerebral small vessel diseases. The main strengths of the trial are described elsewhere.^19^ The additional strengths of the imaging substudies are that they relied on imaging acquired before randomisation (so appearances could not have been affected by allocated treatment), imaging was performed in everyday clinical practice, MRI was done according to a standardised protocol, imaging was collected centrally in DICOM format, and adjudicated by experienced neuroradiologists masked to treatment allocation and outcome using validated rating scales.

The overall characteristics of participants in the trial were similar to those of patients in observational hospital-based studies of antiplatelet therapy use after intracerebral haemorrhage in clinical practice.^5^, ^19^ However, the external validity of our findings can be judged by participants' imaging characteristics, which reflect the inclusion of survivors with haemorrhages that were smaller and had a lower prevalence of subarachnoid and intraventricular extension than those in all-inclusive population-based studies.^2^, ^36^ Therefore, our findings are generalisable to adults who survived a median of 76 days after intracerebral haemorrhage, most of whom had good functional ability and few of whom had a low probability of good functional outcome at 6 months,^19^ in part because of the volumes of their intracerebral haemorrhages, which were smaller than those in population-based studies.^2^, ^36^

The sample size resulted in some small baseline imbalances. The numbers of outcomes were not large enough to detect small or modest differences between subgroups, in particular those brain imaging features that have been proposed to modify the effects of antiplatelet therapy, such as cerebral microbleeds and superficial siderosis.^5^, ^10^, ^13^ However, the extent of heterogeneity in the effects of antiplatelet therapy after intracerebral haemorrhage by brain imaging features was unknown before the trial started, so we could not accurately estimate the sample sizes required to adequately power our subgroup analyses.

In clinical practice, physicians and patients might be reassured by our finding that excluded all but a very modest harmful effect of antiplatelet therapy on recurrent intracerebral haemorrhage in the presence of brain microbleeds. This finding might encourage changes to the current risk-averse approach of not using antiplatelet therapy after intracerebral haemorrhage, driven by findings from a small observational study.^5^, ^9^, ^10^, ^34^ Moreover, there was no strong evidence of heterogeneity between subgroups, and the effect estimates in almost all subgroups were consistent with the trial's overall finding that antiplatelet therapy might reduce the risk of recurrent intracerebral haemorrhage. Furthermore, despite the association between superficial siderosis and recurrent intracerebral haemorrhage,^6^ the effect of antiplatelet therapy on recurrent intracerebral haemorrhage in people with superficial siderosis (HR 0·70, 95% CI 0·17–2·93) might affect clinical equipoise and increase recruitment of people with this imaging feature in future randomised trials of antithrombotic therapies.

The directions and magnitudes of the effects we have found should help to inform the precision of subgroup analyses in imaging substudies in ongoing trials (RESTART-Fr, NCT02966119; and STATICH, NCT03186729) and future randomised controlled trials of antithrombotic therapy after intracerebral haemorrhage. These randomised trials are needed to investigate our findings with greater precision. It is a frequent misconception that risk factors for stroke recurrence in observational studies, such as cerebral microbleeds,^7^, ^32^ are also modifiers of the effects of antithrombotic therapies, although this can only be investigated in randomised controlled trials with larger sample sizes.

Our findings provide the opportunity to estimate the minimum sample size that would be required to demonstrate a potentially statistically significant subgroup interaction with the effects of antiplatelet therapy in this population. If we assume that having more periventricular lucencies causes a four-times greater risk of recurrent intracerebral haemorrhage (HR 0·19 [95% CI 0·04–0·86] for 0–2 periventricular lucencies *vs* 0·89 [0·35–2·23] for 3–4; p_interaction_=0·089), then to detect such an interaction in a future parallel-group randomised trial, assuming similar event rates over 2 years of follow-up, with 90% power at the 5% significance level, a sample size of at least 2200 participants would be needed (or at least 3000 participants at the 1% significance level).

In summary, we excluded all but a very modest harmful effect of antiplatelet therapy on recurrent intracerebral haemorrhage in the presence of brain microbleeds and we did not find strong evidence of heterogeneity in the effects of antiplatelet therapy by other brain imaging features. Further randomised trials are needed to replicate these findings and investigate them with greater precision.

## Data sharing

A fully anonymised version of the dataset used for analysis with individual participant data and a data dictionary will be available for other researchers to apply to use 1 year after publication, via https://datashare.is.ed.ac.uk/handle/10283/3265. Written proposals will be assessed by members of the RESTART trial steering committee and a decision made about the appropriateness of the use of data. A data sharing agreement will be put in place before any data are shared.
