# Supplementary material for: Effects of antiplatelet therapy on stroke risk by brain imaging features of intracerebral haemorrhage and cerebral small vessel diseases: subgroup analyses of the RESTART randomised, open-label trial
Source: Lancet Neurol. 2019 Jul;18(7):643–52. doi: 10.1016/S1474-4422(19)30184-X (PMC7645733; doi:10.1016/S1474-4422(19)30184-X)

THE LANCET
Neurology

Supplementary appendix

This appendix formed part of the original submission and has been peer reviewed. We post it as supplied by the authors.

Supplement to: Al-Shahi Salman R, Minks DP, Mitra D, et al. Effects of antiplatelet therapy on stroke risk by brain imaging features of intracerebral haemorrhage and cerebral small vessel diseases: subgroup analyses of the RESTART randomised, open-label trial. *Lancet Neurol* 2019; published online May 22. http://dx.doi.org/10.1016/S1474-4422(19)30184-X.

Effects of antiplatelet therapy on stroke risk by brain imaging features of intracerebral haemorrhage and cerebral small vessel diseases: subgroup analyses of the RESTART randomised, open-label trial

Online appendix

Appendix of collaborators on delegation logs at hospital sites that recruited at least one participant to RESTART

Sites are listed in descending order of cumulative recruitment (quantified in square brackets), indicating which people took the role of principal investigator (PI).

Edinburgh Royal Infirmary, Edinburgh [39] (Prof. R Al-Shahi Salman (PI), Prof. G Mead, S Burgess, C Lerpiniere, R O'Brien, R Paulton, F Doubal, K McCormick, N Hunter, P Taylor, R Parakramawansha, J Perry, G Blair, A MacRaild); **Salford Royal Foundation Trust, Manchester [21]** (A Parry-Jones (PI), K Shaw, I Burger, A Ingham, T Marsden, J Morell, Z Naing, J Perez, A Hall, R Jarapa, E Wood, V O'Loughlin, S Marshall, L Harrison, M Punter, S Lee, M Johnes); **Northwick Park Hospital, Harrow [18]** (D Cohen (PI), S Davies, K Njoku, M Mpelembue, L Burgess, R Licenik, M Ngwako, N Nisar, R Niranchanan, T Roganova, R Bathula, J Devine, A David, A Oshodi, F Guo, M Abbdul-saheb, A Chandrakumar, A Chamberlain, R Ballantine, E Owoyele, V Sukdeo, P Poku); **Royal Hallamshire Hospital, Sheffield [13]** (K Harkness (PI), C Blank (PI), P Bayliss, E Richards, K Birchall, O Balitska, A Ali, F Kibutu, C Doyle, J Howe, C Kamara, K Stocks, Prof. A Majid, A Maatouk, L Barron, R Lindert, J Redgrave, K Dakin); **Torbay District General Hospital, Torquay [13]** (B Bhaskaran (PI), S Szabo, I Salih, D Kelly, D Tomlin, H Bearne, P Fitzell, J Buxton, G Ayres, H Bhakri, J Garfield-Smith, K Horan, A Saulat); **Southend University Hospital NHS Foundation Trust, Westcliff on Sea [12]** (P Guyler (PI), D Sinha (PI) T Loganathan, A Siddiqui, L Coward, S Tysoe, S Kunhunny, S Shah, K Ng, N Menon, R Orath Prabakaran, S Kelavkar, S Rashmi, D Ngo); **Monklands Hospital, Airdrie [11]** (M Barber (PI), D Esson, F Brodie); **Morriston Hospital, Swansea [11]** (T Anjum (PI), M Wani (PI), M Krishnan, L Quinn, J Spencer, S Chenna, S Storton, T Jones, H Thompson-Jones, L Dacey, S Thomas, T Beaty, S Treadwell, C Davies, L Connor, S Tucker, G Gainard, P Slade); **University Hospitals of North Midlands NHS Trust, Stoke-on-Trent [11]** (G Muddegowda (PI), R Sanyal (PI), S Stevens, A Butler, R Varquez, A Remegoso, N Abano, F Alipio, H Denic, R Carpio, C Causley, A Moores, S Lyjko, Prof. C Roffe, J Hiden, P Ferdinand, A Barry, H Maguire, J Grocott, K Finney); **Victoria Hospital, Kirkcaldy [11]** (V Cvoro (PI), M Couser, K Ullah, N Chapman, K McCormick, S Mcauley, S Pound); **City Hospital, Nottingham [10]** (S Raghunathan (PI), F Shelton, A Hedstrom, N Gilzeane, J Roffe, J Clarke, D Havard, A Buck, K Krishnan, M Godfrey, N Sprigg, S Sheikh, K Whittamore, R Keshvara, B Jackson, J Appleton, Z Law, O Matias, G Wilkes, C Jordan); **Hillingdon Hospital, Uxbridge [10]** (E Vasileiadis (PI), C Mason, A Parry, G Landers, M Holden, B Aweid); **Yeovil District Hospital, Yeovil [10]** (K Rashed (PI), L Balian, C Vickers, B Williams-Yesson, E Keeling, S Board, J Allison, C Buckley, J Board, D Wood, T Pitt-Kerby, A Tanate); **Doncaster Royal Infirmary, Doncaster [9]** (M Kini (PI), D Walstow, D Chadha, R Fong); **North Middlesex University Hospital, London [9]** (R Luder (PI), T Adesina, J Gallagher, M Bhargava, C van Someren, E Murali, H Bridger); **Royal Cornwall Hospital,**

Truro [9] (F Harrington (PI), A James, K Adie, A Mate, G Courtauld, C Schofield, K Bond, L Lucas, B Maund, S Ellis); **Royal Devon & Exeter Hospital, Exeter [9]** (P Mudd (PI), M James, S Keenan, A Bowring, J Cageao, D Strain, H Kingwell, C Roughan, A Hemsley, J Sword, K Miller, A Goff, K Gupwell, K Thorpe); **Royal Preston Hospital, Preston [9]** (H Emsley (PI), S Punekar (PI), A McLoughlin, S Sultan, B Gregory, S Raj, D Doyle); **Queen Elizabeth University Hospital, Glasgow [9]** (Prof. K Muir (PI), W Smith, N Day, A Welch, F Moreton, B Cheripelli, D Kalladka, X Huang, S El Tawil, S Ramachandran, C Crosbie, J Elliot); **Guys & St Thomas, London [8]** (Prof. T Rudd (PI), A Bhalla, J Birns, K Marks, S Kullane); **Southampton General Hospital, Southampton [8]** (N Weir (PI), C Allen, V Pressly, E Battersby-Wood, P Crawford, S Egerton, A Blades, G Howard, J Marigold, S Evans, A Walters, F Smith, I Gartrell, C Cox, R Creeden, S Smith, C Boxall); **Ystrad Mynach Hospital, Ystrad Mynach, Newport [8]** (J Hewitt (PI), C Nott, S Procter, S Buckle, J Whiteman, C Triscott, R Mardania, R Wallace, J Gray); **Calderdale Royal Hospital, Halifax [7]** (A Nair (PI), J Greig, P Rana, M Robinson, M Alam); **University College London Hospital, London [7]** (Prof D Werring (PI), I Jones, A Banaras, L Crook, C Watchurst, M Brezitski, K Patel, D Wilson, R Erande, C Hogan, N Oji, N Francia, A Ashton, S Feerick, I Hostettler, T Al-Mayhani, E Elliott); **Altnagelvin Hospital, Derry/Londonderry [6]** (M McCarron (PI), J McKee, M Doherty, F McVerry, C Blair); **Bristol Royal Infirmary, Bristol [6]** (C Holmes (PI), S Caine (PI), M Osborn, E Dodd, P Murphy, N Devitt, P Baker, A Steele, L Guthrie, S Clarke); **Gloucestershire Royal Hospital, Gloucester [6]** (D Dutta (PI), P Brown, D Ward, F Davis, J Turfrey, R Bakawala, C Hughes, K Collins, S O'Connell, J Glass); **James Cook University Hospital, Middlesbrough [6]** (D Broughton (PI), D Tryambake (PI), L Dixon, K Chapman, A Young, A Bergin, A Sigsworth); **Kings Mill Hospital, Mansfield [6]** (M Cooper (PI), M Nasar, I Wynter, A Rajapakse); **Leeds General Infirmary, Leeds [6]** (A Hassan (PI), M Kambafwile, L Makawa, D Waugh, E Veraque, M Randall, V Papavasileiou); **Royal Liverpool and Broadgreen University Hospital, Liverpool [6]** (A Manoj (PI), M Wilkinson, G Fletcher, P Lopez, P Cox, P Fitzsimmons, N Sharma); **Royal United Hospital, Bath [6]** (J Choulerton (PI), B Madigan, D Button, L Dow, L Gbadamoshi, J Avis, S McCann, L Shaw, D Howcroft, S Lucas, A Stone); **St Georges Healthcare NHS Trust, London [6]** (G Cluckie (PI), C Lovelock (PI), B Patel, B Clarke, N Chopra, K Kennedy, R Williams, L Kerin, N Jeyaraj, L Choy, N Clarke, F Watson, S Trippier, B Moynihan, U Khan, N Dayal, C Orefo, T Adedoyin, R Ghatala, A Blight, V Jones, J O'Reilly); **The Royal Bournemouth Hospital, Bournemouth [6]** (K Thavanesan (PI), D Tiwari (PI), C Cox, J Roberts, B Jupp, M Keltos, A Iqbal, C Bagnall, L Tucker, A Ljubez, O David, E Rogers, C Ovington, J Bell, B Longland, G Hann); **University Hospital Aintree, Liverpool [6]** (C Cullen (PI), H Thant, T Ingram, M Zoe, J Peters, V Sutton, R Durairaj, D Shackcloth, J Ewing, S Stevenson, M Harrison); **University Hospital of North Tees, Stockton [6]** (I Anwar (PI), B Kumar, H Skinner, T Nozedar, D McArdle, S Crawford, A Annamalai, A Ramshaw); **Western General Hospital, Edinburgh [6]** (Prof. M Dennis (PI), Prof. C Sudlow, W

Whiteley, C Lerpiniere, Prof. R Al-Shahi Salman, R Fraser); **Aberdeen Royal Infirmary, Aberdeen [5]** (M MacLeod (PI), J Irvine, A Joyson, H Gow, J Furnace, B Jagpal, S Ross, S Nelson, R Clarke, N Crouch, K Klaasen, B MacLennan, V Taylor); **Addenbrooke's Hospital, Cambridge [5]** (E O'Brien (PI), S Finlay, H Hayhoe, D Handley, S Kelly, J Francis, N Hannon, G Zachariah, J Mcgee, J Mitchell, E Amis, J Sesay, S Crisp); **Barnet Hospital, Barnet [5]** (D Epstein (PI), A Shukla, I Jones, V Krishnamurthy, P Nicholas, A Webber, S Qureshi, J Penge); **Bradford Royal Infirmary, Bradford [5]** (H Ramadan (PI), S Maguire, C Patterson, R Bellfield, B Hairsine, O Quinn, M Hooley, K Stewart); **John Radcliffe Hospital, Oxford [5]** (U Schulz (PI), R Teal, P Mathieson, I Reckless, J Kennedy, Prof. G Ford, G Lenti, G Harston); **Nevill Hall Hospital, Abergavenny [5]** (B Richard (PI), S Buckle, S Procter, S Moseley, C Nott, J Whiteman, C Triscott, R Wallace, M Edwards, H Lawson, M Tayler); **The Royal London Hospital, London [5]** (T Harrison (PI), K Saastamoinen (PI), A Salek-Haddadi, D Hove, L Howaniec, G Grimwood, O Redjep, F Humphries, S Amlani, L Cuenoud, E Erumere, G Auld, L Argandona); **University Hospital North Durham, Durham [5]** (Y Pai (PI), M Dhakal (PI), S Dima (PI), B Esisi (PI), G Smith, M Garside, D Bruce, R Hayman, S Clayton, E Brown, G Rogers, M Naeem, V Baliga); **University Hospital of Wales, Cardiff [5]** (T Hughes (PI), B Morse, S White, S Schwarz, E Tallantyre, A Osman, H De Berker, B Jelley); **Wythenshawe Hospital, Manchester [5]** (E Gamble (PI), B Charles, R Grue, A Chaudhry, S Blane, A Hague, C Lambert); **Ayr Hospital, Ayr [4]** (S Ghosh (PI), D Gilmour, E Barrie, M Henry); **Charing Cross Hospital, London [4]** (M Venter (PI), A Kar (PI), S Mashate, K Harvey, L Gardener, V Nguyen, B Hazel, O Geraghty, O Halse, P Wilding, V Tilley); **Derby Royal Hospital, Derby [4]** (Prof. T England (PI), A Hedstrom, M Maddula, Prof. R Donnelly); **Heartlands Hospital, Birmingham [4]** (R Yadava (PI), K Azhar (PI), M Sangombe, J Reddan, S Stafford); **New Cross Hospital, Wolverhampton [4]** (K Fotherby (PI), D Morgan, F Baig, K Jennings-Preece, D Butler, N Ahmad, B Rai, A Stevens, A Willberry); **Queen Alexandra Hospital, Portsmouth [4]** (P Siddegowda (PI), L Hyatt, A Saulat, J Tandy, P Howard, T Dobson, D Jarrett, S Ponnambath, S Valentine, C James, R Butler, Y Harrington-Davies, A Suttling); **Queen Elizabeth Hospital, Gateshead [4]** (B Esisi (PI), T Cassidy (PI), B McClelland, M Bokhari); **Raigmore Hospital, Inverness [4]** (P Findlay (PI), A Macaden, I Shread, C Barr); **Royal Infirmary, Glasgow [4]** (Prof. P Langhorne (PI), G Kerr, F Wright, R Graham, C McAlpine, L Humphreys, M Iqbal); **Royal Surrey County Hospital, Guildford [4]** (K Pasco (PI), O Balazikova, A Nasim, C Peixoto, S Shahmehri, L Gallagher); **William Harvey Hospital, Ashford [4]** (T Webb (PI), L Cowie, A Thomson, H Rudenko, A Verrion, E Beranova, T Cosier, S Walker, S McDonald, N Schumacher); **Derriford Hospital, Plymouth [3]** (A Mohd Nor (PI), C Eglinton, N Persad, C Brown, M Weinling, A Shah, J Baker, B Hyams); **Forth Valley Royal Hospital, Larbert [3]** (A Byrne (PI), C McGhee, A Smart, C Copeland); **Hull Royal Infirmary, Hull [3]** (R Rayessa (PI), L Wilson, C Naylor, A Rodgers, S Wilson, E Clarkson); **Pinderfields Hospital, Wakefield [3]** (M Carpenter (PI), M Walker, R Davey, A

Needle, R Fathima, G Bateman, A Stanners, P Datta, L Jackson, J Ball); **Royal Victoria Infirmary, Newcastle upon Tyne [3]** (M Davis (PI), H Guy, N Atkinson, M Fawcett, T Thompson, C Hays, S Woodward, V Hogg); **Salisbury District Hospital, Salisbury [3]** (T Black (PI), A Anthony, S Miriam, C Clarke, D Mead, M Tribbeck, J Cronin, R Fennelly); **St Mary's Hospital, Newport Isle of Wight [3]** (M Haque (PI), E Hakim (PI), S Symonds, M Maanoosi, J Herman); **St Richards Hospital, Chichester [3]** (S Ivatts (PI), Y Baird, M Sally, I Amey, L Clayton- Evans, S Newton, I Chadbourn); **Victoria Hospital, Blackpool [3]** (J McIlmoyle (PI), C Jeffs, C Dickinson, J Howard, S Anwar, S Dhar, K Jones, M Siddiq, C Clay); **Arrowe Park Hospital, Wirral [2]** (R Davies (PI), P Owings, G Sangster, V Gott, V Little, P Weir, S Cherian, D Jose, H Moroney, S Downham, A Dodd, L Codd, V Vettimootal Johnson, N Robinson); **Barnsley Hospital NHS Foundation Trust, Barnsley [2]** (A Ahmed (PI), M Albazzaz (PI), S Johnson, C Denniss, T Zahoor, M Cunningham); **Countess of Chester NHS Foundation Trust, Chester [2]** (T Webster (PI), K Chatterjee, A Nallasivan, S Haider, S Leason, C Perkins, S Seagrave); **Hereford County Hospital, Hereford [2]** (C Jenkins (PI), F Price, C Hughes, L Mercer); **Leicester Royal Infirmary, Leicester [2]** (D Eveson (PI), A Mistri, L Manning, C Patel, M Moqsith, S Khan, C Stephens, S Sattar, M Lam, K Musarrat); **Leighton Hospital, Crewe [2]** (L Kalathil (PI), R Miller, M Salehin, N Gautam, D Bailey, K Amor, J Meir); **Luton & Dunstable NHSFT University Hospital, Luton [2]** (L Sekaran (PI), F Justin, M Tate, K Bharaj, R Simon, N Mohammed, S Sethuraman, D Phiri, M Chauhan); **Musgrove Park Hospital, Taunton [2]** (M Hussain (PI), S Brown, M Harvey, R Whiting, M Khan, J Homan, L Foote, N Hunt, A Whitcher, C Pawley, E Foster, J Foot, H Durman, L Brotherton); **Norfolk & Norwich University Hospital, Norwich [2]** (K Metcalf (PI), J Jagger, S McDonald, K Waterfield, P Sutton, J Saada, A Wiltshire, R Perfitt, R Greenwood, N Shinh, A Anversha, G Ravenhill); **Pilgrim Hospital, Boston [2]** (D Mangion (PI), S Markova, A Hardwick, T Lawrence, J Fletcher, C Constantin, K Pettitt, I Thomas); **Queens Hospital, Romford [2]** (S Andole (PI), N Gadapa, K Dunne, M Krommyda, E Burssens, C Plewa, S King); **Royal Hampshire County Hospital, Winchester [2]** (N Smyth (PI), J Wilson, E Giallombardo, C Eglinton, L Sykes); **Royal Lancaster Infirmary, Lancaster [2]** (P Kumar (PI), P Thomas, l Dunn, C Culmsee, I Huggett, J Barker); **Royal Victoria Hospital, Belfast [2]** (I Wiggam (PI), A Wallace, E Kerr, A Fulton, A Hunter, S Tauro, S Cuddy); **Solihull Hospital, Solihull [2]** (K Elfandi (PI), U Khan, S Stafford, J Reddan); **Sunderland Royal Hospital, Sunderland [2]** (M Myint (PI), R O'Brien (PI), H Brew, N Majmudar, J OConnell, G Bunea, C Fox, D Gulliver, N Sattar, B Mokoena, A Smith, E Osborne, R Krishnamurthy); **Ulster Hospital, Belfast [2]** (D Wilson (PI), B Wroath, K Dynan, M Power, S Thompson, V Adell); **West Cumberland Hospital, Whitehaven [2]** (E Orugun (PI), U Poultney, H Crowther, R Glover, S Thornthwaite); **West Suffolk Hospital, Bury St Edmunds [2]** (A Nicolson (PI), L Wood, J Imam, J White); **Bedford Hospital, Bedford [1]** (H Ni (PI), C Graham, B Rahman, J Milligan, J Jose); **Chesterfield Royal Hospital, Chesterfield [1]** (M Sajid (PI), G Ghaly, M Ball, R Gascoyne); **Dorset County**

Hospital NHS Foundation Trust, Dorchester [1] (H Proeschel (PI), S Sharpe, S Horton, S Jones, E Beaves); **Epsom General Hospital, Epsom [1]** (J Putterill (PI), R Jha, R Gallifent, P Kakar); **Hairmyres Hospital, East Kilbride [1]** (B Yip (PI), M Bell, B MacInnes, L MacLiver, D Esson); **Lister Hospital, Stevenage [1]** (A Pusalkar (PI), K Chan, P Dangri, K Crabtree, H Beadle, A Cook); **Peterborough City Hospital, Peterborough [1]** (S Subramonian (PI), P Owusu-Agyei (PI), N Temple, N Butterworth-Cowin); **Poole Hospital, Poole [1]** (S Ragab (PI), K Knops, E Jinks, C Dickson, L Gleave, J Leggett, J Dube, T Garcia); **Prince Charles Hospital, Merthyr Tydfil [1]** (R Dewar (PI), K Thomas, J White); **Queen Elizabeth Hospital, Birmingham [1]** (D Sims (PI), J Hurley, M Willmot, C Sutton, E Littleton, S Maiden, J Cunningham, R Jones, C Green, M Bates); **Queen Elizabeth Hospital, Kings Lynn [1]** (R Shekhar (PI), R Crown, E Gilham, T Fuller, I Ahmed, K Waterfield); **Royal Blackburn Hospital, Blackburn [1]** (N Goorah (PI), A Bell, C Kelly, A Singh, J Walford, S Duberley, B Tomlinson, F Patel); **Royal Sussex County Hospital, Brighton [1]** (I Kane (PI), N Gainsborough, J Gaylard, J Breeds, Prof. C Rajkumar, S Hervey, A Pitt-Ford, L Latter, E Barbon, P Thompson); **Sandwell General Hospital, Birmingham [1]** (S Ispoglou (PI), R Evans, S Ankolekar, A Hayes); **South West Acute Hospital, Enniskillen [1]** (B Keegan (PI), M Doherty, J Kelly, C Blair); **Stepping Hill Hospital, Stockport [1]** (S Krishnamoorthy (PI), J Vassallo, D Walter, H Cochrane); **The Princess Royal Hospital, Telford [1]** (M Srinivasan (PI), F Hurford, D Donaldson, R Campbell, N Motherwell, I Mukherjee); **University Hospitals Coventry and Warwickshire, Coventry [1]** (A Kenton (PI), S Nyabadza, I Martin, B Hunt, H Hassan, B Dallol, S O'Toole).

Literature search strategies used to put the research in context

MEDLINE search strategy

1. exp Cerebral Hemorrhage/
2. ((brain$ or cerebr$ or cerebell$ or intracerebral or intracran$ or parenchymal or intraparenchymal or intraventricular or infratentorial or supratentorial or basal gangli$ or putaminal or putamen or posterior fossa or hemispher$) adj5 (h?emorrhag$ or h?ematoma$ or bleed$)).tw.
3. ((h?emorrhag$ or bleed$) adj5 (stroke or apoplex$)).tw.
4. (ICH or ICHs).tw.
5. 1 or 2 or 3 or 4
6. Randomized Controlled Trials/
7. random allocation/
8. Controlled Clinical Trial/
9. control groups/
10. clinical trials/ or clinical trials, phase i/ or clinical trials, phase ii/ or clinical trials, phase iii/ or clinical trials, phase iv/
11. double-blind method/
12. single-blind method/
13. Placebos/
14. placebo effect/
15. randomi$ed controlled trial.mp.
16. controlled clinical trial.pt.
17. (clinical trial or clinical trial phase i or clinical trial phase ii or clinical trial phase iii or clinical trial phase iv).pt.
18. (random$ or RCT or RCTs).tw.
19. (controlled adj5 (trial$ or stud$)).tw.
20. (clinical$ adj5 trial$).tw.
21. ((control or treatment or experiment$ or intervention) adj5 (group$ or subject$ or patient$)).tw.
22. (quasi-random$ or quasi random$ or pseudo-random$ or pseudo random$).tw.
23. ((singl$ or doubl$ or tripl$ or trebl$) adj5 (blind$ or mask$)).tw.
24. (placebo$ or sham).tw.

25. ((control or experiment$ or conservative) adj5 (treatment or therapy or procedure or manage$)).tw.
26. trial.ti.
27. (assign$ or allocat$).tw.
28. or/6-27
29. exp amyloid/
30. exp Cerebral Amyloid Angiopathy/
31. exp Amyloid beta-Peptides/
32. cerebral amyloid angiopathy.mp.
33. CAA.mp.
34. white matter lesion$.mp.
35. WML.mp.
36. white matter disease$.mp.
37. white matter hyperintensit$.mp.
38. WMH.mp.
39. leu?oaraiosis.mp.
40. exp Microcirculation/
41. lacun$.mp.
42. lacunar infarct$.mp.
43. exp Stroke, Lacunar/
44. small vessel$.mp.
45. small infarct$.mp.
46. microinfarct$.mp.
47. subcortical lesion$.mp.
48. subcortical infarct$.mp.
49. microvascular$.mp.
50. microbleed$.mp.
51. micro-bleed$.mp.
52. microh?emorrhage$.mp.
53. micro-h?emorrhage$.mp.
54. perivascular space$.mp.

55. PVS.mp.
56. enlarged perivascular space$.mp.
57. EPVS.mp.
58. virchow-Robin space$.mp.
59. VRS.mp.
60. small vessel disease$.mp.
61. SVD.mp.
62. exp arteriolosclerosis/
63. exp Arteriosclerosis/
64. (atherosclerosis adj3 small).mp.
65. fibrinoid necrosis.mp.
66. lipohyalinosis.mp.
67. microatheroma.mp.
68. microaneurysm$.mp.
69. venous collagenosis.mp.
70. cerebral vascu$.mp.
71. cerebrovasc$.mp.
72. exp Cerebrovascular Circulation/
73. exp Cerebrovascular Disorders/
74. microscopic bleed$.mp.
75. microscopic hemorrhag$.mp.
76. microscopic haemorrhag$.mp.
77. dot like h?emosiderin spot.mp.
78. Dot-like h?emosiderin spot.mp.
79. Multifocal signal loss.mp.
80. Hypointense lesion.mp.
81. Hypointense foci.mp.
82. Lacunar hemorrhag$.mp.
83. Lacunar haemorrhag$.mp.
84. minute hemorrhag$.mp.

85. minute haemorrhag$.mp.

86. superficial siderosis.mp.

87. cSS.mp.

88. exp Brain/

89. cranial.mp.

90. cerebr$.mp.

91. cerebell$.mp.

92. brain$.mp.

93. intracerebral.mp.

94. intracranial.mp.

95. hemispher$.mp.

96. infratentorial.mp.

97. supratentorial.mp.

98. or/88-97

99. or/29-87

100. 98 and 99

101. exp anticoagulants/

102. exp Vitamin K/ai or thrombin/ai or factor Xa/ai or exp Blood coagulation factors/ai

103. exp antithrombins/ or hirudin therapy/

104. (anticoagul$ or antithromb$).tw.

105. (Vitamin K antagonist$ or VKA or VKAs).tw.

106. (direct$ adj3 thrombin adj3 inhib$).tw.

107. DTI$1.tw.

108. ((factor Xa or factor 10a or fXa or autoprothrombin c or thrombokinase) adj3 inhib$).tw.

109. (activated adj3 (factor X or factor 10) adj3 inhib$).tw.

110. (acenocoumarol$ or dicoumarol$ or ethyl biscoumacetate$ or phenprocoumon$ or warfarin$ or ancrod$ or citric acid$ or coumarin$ or chromonar$ or coumestro$ or esculi$ or ochratoxin$ or umbelliferone$ or dermatan sulfate$ or dextran$ or edetic acid$ or enoxaparin$ or gabexate$ or heparin$ or lmwh$ or nadroparin$ or pentosan sulfuric polyester$ or phenindione$ or protein c or protein s or tedelparin$).tw,nm.

111. (tinzaparin or parnaparin or dalteparin or reviparin or danaparoid or lomoparan or org 10172 or mesoglycan or polysaccharide sulphate$ or sp54 or sp-54 or md805 or md-805 or cy222 or cy-222 or cy216 or cy-216).tw,nm.
112. (Marevan or Fragmin$ or Fraxiparin$ or Klexane).tw,nm.
113. (argatroban or MD805 or MD-805 or dabigatran or ximelagatran or melagatran or efegatran or flovagatran or inogatran or napsagatran or bivalirudin or lepirudin or hirudin$ or desirudin or desulfatohirudin or hirugen or hirulog or AZD0837 or bothrojaracin or odiparcil).tw,nm.
114. (xabans or antistasin or apixaban or betrixaban or du 176b or eribaxaban or fondaparinux or idraparinux or otamixaban or razaxaban or rivaroxaban or edoxaban or yagin or ym 150 or ym150 or LY517717).tw,nm.
115. exp platelet aggregation inhibitors/ or exp platelet glycoprotein gpiib-iiia complex/ai
116. (antiplatelet$ or anti-platelet$ or antiaggreg$ or anti-aggreg$ or (platelet$ adj3 inhibit$) or (thrombocyt$ adj3 inhibit$)).tw.
117. (alprostadil$ or aspirin$ or acetylsalicylic acid or acetyl salicylic acid$ or acetyl?salicylic acid or epoprostenol$ or ketanserin$ or ketorolac tromethamine$ or milrinone$ or mopidamol$ or procainamide$ or thiophen$ or trapidil$ or picotamide$ or ligustrazine$ or levamisol$ or suloctidil$ or ozagrel$ or oky046 or oky-046 or defibrotide$ or cilostazol or satigrel or sarpolgrelate or kbt3022 or kbt-3022 or isbogrel or cv4151 or cv-4151 or ((glycoprotein iib$ or gp iib$) adj5 (antagonist$ or inhibitor$)) or GR144053 or GR-144053 or triflusal).tw,nm.
118. (Beraprost or Cicaprost or Cilostazol or Clopidogrel or Dipyridamole or Iloprost or Indobufen or Lepirudin or Pentosan Polysulfate or Pentoxifylline or Piracetam or Prostacyclin or Sulfinpyrazone or Sulphinpyrazone or Ticlopidine or Triflusal or Abciximab or Disintegrin or Echistatin or Eptifibatide or Lamifiban or Orbofiban or Roxifiban or Sibrafiban or Tirofiban or Xemilofiban or terutroban or picotamide or prasugrel).tw,nm.
119. (Dispril or Albyl$ or Ticlid$ or Persantin$ or Plavix or ReoPro or Integrilin$ or Aggrastat).tw,nm.
120. or/101-119
121. exp Tomography, X-Ray Computed/
122. exp Magnetic Resonance Imaging/
123. 121 or 122
124. 5 and 28 and 100 and 120 and 123
125. exp animals/ not humans/
126. 124 not 125

EMBASE (Ovid) search strategy

1. exp amyloid/
2. exp vascular amyloidosis/
3. exp amyloid protein/
4. exp amyloid beta protein/
5. cerebral amyloid angiopathy.mp.
6. CAA.mp.
7. exp leukoaraiosis/
8. exp white matter lesion/
9. WML.mp.
10. white matter disease$.mp.
11. white matter hyperintensit$.mp.
12. WMH.mp.
13. exp brain microcirculation/
14. lacun$.mp.
15. lacunar infarct$.mp.
16. small vessel$.mp.
17. small infarct$.mp.
18. microinfarct$.mp.
19. subcortical lesion$.mp.
20. subcortical infarct$.mp.
21. microvascular$.mp.
22. microbleed$.mp.
23. micro-bleed$.mp.
24. microh?emorrhage$.mp.
25. micro-h?emorrhage$.mp.
26. perivascular space$.mp.
27. PVS.mp.
28. enlarged perivascular space$.mp.
29. EPVS.mp.

30. virchow-Robin space$.mp.
31. VRS.mp.
32. small vessel disease$.mp.
33. SVD.mp.
34. exp arteriolosclerosis/
35. exp microaneurysm/
36. (atherosclerosis adj3 small).mp.
37. fibrinoid necrosis.mp.
38. lipohyalinosis.mp.
39. microatheroma.mp.
40. venous collagenosis.mp.
41. microscopic bleed$.mp.
42. microscopic hemorrhag$.mp.
43. microscopic haemorrhag$.mp.
44. dot like h?emosiderin spot.mp.
45. Dot-like h?emosiderin spot.mp.
46. Multifocal signal loss.mp.
47. Hypointense lesion.mp.
48. Hypointense foci.mp.
49. Lacunar hemorrhag$.mp.
50. Lacunar haemorrhag$.mp.
51. minute hemorrhag$.mp.
52. minute haemorrhag$.mp.
53. superficial siderosis.mp.
54. cSS.mp.
55. exp Brain/
56. cranial.mp.
57. cerebr$.mp.
58. cerebell$.mp.
59. brain$.mp.

60. intracerebral.mp.
61. intracranial.mp.
62. hemispher$.mp.
63. infratentorial.mp.
64. supratentorial.mp.
65. or/55-64
66. or/1-54
67. 65 and 66
68. *basal ganglion hemorrhage/ or *brain hemorrhage/ or *brain ventricle hemorrhage/ or *cerebellum hemorrhage/
69. ((brain$ or cerebr$ or cerebell$ or intracerebral or intracran$ or parenchymal or intraparenchymal or intraventricular or infratentorial or supratentorial or basal gangli$ or putaminal or putamen or posterior fossa or hemispher$ or stroke or apoplex$) adj5 (h?emorrhag$ or h?ematoma$ or bleed$)).ti.
70. 68 or 69
71. (ICH or ICHs).ti.
72. 70 or 71
73. randomized controlled trial/ or "randomized controlled trial (topic)"/
74. Randomization/
75. Controlled Study/
76. control group/
77. clinical trial/ or phase 1 clinical trial/ or phase 2 clinical trial/ or phase 3 clinical trial/ or phase 4 clinical trial/ or controlled clinical trial/
78. Double Blind Procedure/
79. Single Blind Procedure/ or triple blind procedure/
80. placebo/
81. drug comparison/ or drug dose comparison/
82. random$.tw.
83. (controlled adj5 (trial$ or stud$)).tw.
84. (clinical$ adj5 trial$).tw.
85. ((control or treatment or experiment$ or intervention or surgical) adj5 (group$ or subject$ or patient$)).tw.
86. ((singl$ or doubl$ or tripl$ or trebl$) adj5 (blind$ or mask$)).tw.

87. or/73-86

88. anticoagulant agent/ or antivitamin k/ or exp blood clotting inhibitor/ or exp coumarin anticoagulant/ or defibrotide/ or dextran sulfate/ or fluindione/ or glycosaminoglycan polysulfate/ or exp heparin derivative/ or lupus anticoagulant/ or phenindione/

89. (anticoagul$ or antithromb$).tw.

90. (Vitamin K antagonist$ or VKA or VKAs).tw.

91. (direct$ adj5 thrombin adj5 inhib$).tw.

92. DTI$1.tw.

93. ((factor Xa or factor 10a or fXa or autoprothrombin c or thrombokinase) adj5 inhib$).tw.

94. (activated adj5 (factor X or factor 10) adj5 inhib$).tw.

95. (acenocoumarol$ or dicoumarol$ or ethyl biscoumacetate$ or phenprocoumon$ or warfarin$ or ancrod$ or citric acid$ or coumarin$ or chromonar$ or coumestro$ or esculi$ or ochratoxin$ or umbelliferone$ or dermatan sulfate$ or dextran$ or edetic acid$ or enoxaparin$ or gabexate$ or heparin$ or lmwh$ or nadroparin$ or pentosan sulfuric polyester$ or phenindione$ or protein c or protein s or tedelparin$).tw.

96. (tinzaparin or parnaparin or dalteparin or reviparin or danaparoid or lomoparan or org 10172 or mesoglycan or polysaccharide sulphate$ or sp54 or sp-54 or md805 or md-805 or cy222 or cy-222 or cy216 or cy-216).tw.

97. (Marevan or Fragmin$ or Fraxiparin$ or Klexane).tw.

98. (argatroban or MD805 or MD-805 or dabigatran or ximelagatran or melagatran or efegatran or flovagatran or inogatran or napsagatran or bivalirudin or lepirudin or hirudin$ or desirudin or desulfatohirudin or hirugen or hirulog or AZD0837 or bothrojaracin or odiparcil).tw.

99. (xabans or antistasin or apixaban or betrixaban or du 176b or eribaxaban or fondaparinux or idraparinux or otamixaban or razaxaban or rivaroxaban or yagin or ym 150 or ym150 or LY517717).tw.

100. 88 or 89 or 90 or 91 or 92 or 93 or 94 or 95 or 96 or 97 or 98 or 99

101. exp antithrombocytic agent/

102. fibrinogen receptor/dt [Drug Therapy]

103. (antiplatelet$ or anti-platelet$ or antiaggreg$ or anti-aggreg$ or (platelet$ adj5 inhibit$) or (thrombocyt$ adj5 inhibit$)).tw.

104. (alprostadil$ or aspirin$ or acetylsalicylic acid or acetyl salicylic acid$ or acetyl?salicylic acid or epoprostenol$ or ketanserin$ or ketorolac tromethamine$ or milrinone$ or mopidamol$ or procainamide$ or thiophen$ or trapidil$ or picotamide$ or ligustrazine$ or levamisol$ or suloctidil$ or ozagrel$ or oky046 or oky-046 or defibrotide$ or

cilostazol or satigrel or sarpolgrelate or kbt3022 or kbt-3022 or isbogrel or cv4151 or cv-4151 or ((glycoprotein iib$ or gp iib$) adj5 (antagonist$ or inhibitor$)) or GR144053 or GR-144053 or triflusal).tw.

105. (Argatroban or Beraprost or Cicaprost or Cilostazol or Clopidogrel or Dipyridamole or Iloprost or Indobufen or Lepirudin or Pentosan Polysulfate or Pentoxifylline or Piracetam or Prostacyclin or Sulfinpyrazone or Sulphinpyrazone or Ticlopidine or Triflusal or Abciximab or Disintegrin or Echistatin or Eptifibatide or Lamifiban or Orbofiban or Roxifiban or Sibrafiban or Tirofiban or Xemilofiban or terutroban or picotamide or prasugrel).tw.
106. (Dispril or Albyl$ or Ticlid$ or Persantin$ or Plavix or ReoPro or Integrilin$ or Aggrastat).tw.
107. 101 or 102 or 103 or 104 or 105 or 106
108. 100 or 107
109. exp computer assisted tomography/
110. exp nuclear magnetic resonance imaging/
111. 109 or 110
112. 67 and 72 and 87 and 108 and 111
113. exp animals/ not humans/
114. 112 not 113

Exploratory sub-group analyses of the risk of first ischaemic stroke (the secondary outcome) by CT biomarkers

Subgroup	Start antiplatelet drugs [Events/N(%)]	Avoid antiplatelet drugs [Events/N(%)]	Adjusted Hazard Ratio (95% CI)	Test of interaction p-value
Largest intracerebral haemorrhage location				
Lobar	7/100 (7.0%)	10/102 (9.8%)	0.62 (0.24 to 1.65)	.699
Other	11/152 (7.2%)	14/152 (9.2%)	0.80 (0.36 to 1.77)	
Prior vascular lesions				
Present	16/154 (10.4%)	17/162 (10.5%)	1.02 (0.51 to 2.01)	.095
Absent	2/98 (2.0%)	7/92 (7.6%)	0.24 (0.05 to 1.13)	
Periventricular lucencies				
0-2	10/165 (6.1%)	14/146 (9.6%)	0.57 (0.25 to 1.29)	.328
3-4	8/87 (9.2%)	10/108 (9.3%)	1.06 (0.42 to 2.70)	
Atrophy				
0-2	16/215 (7.4%)	16/211 (7.6%)	0.95 (0.48 to 1.91)	.149
3-4	2/37 (5.4%)	8/43 (18.6%)	0.27 (0.06 to 1.29)	
Edinburgh CT-only criteria for acute intracerebral haemorrhages with lobar epicentres				
Non-lobar intracerebral haemorrhage	11/152 (7.2%)	14/152 (9.2%)	0.80 (0.36 to 1.75)	.603
Lower probablility of cerebral amyloid angiopathy	7/83 (8.4%)	7/82 (8.5%)	0.91 (0.32 to 2.59)	
High probablility of cerebral amyloid angiopathy	0/14 (0.0%)	2/15 (13.3%)	0.16 (0.001 to 2.02)	
Overall	18/252 (7.1%)	24/254 (9.4%)	0.73 (0.39 to 1.34)	

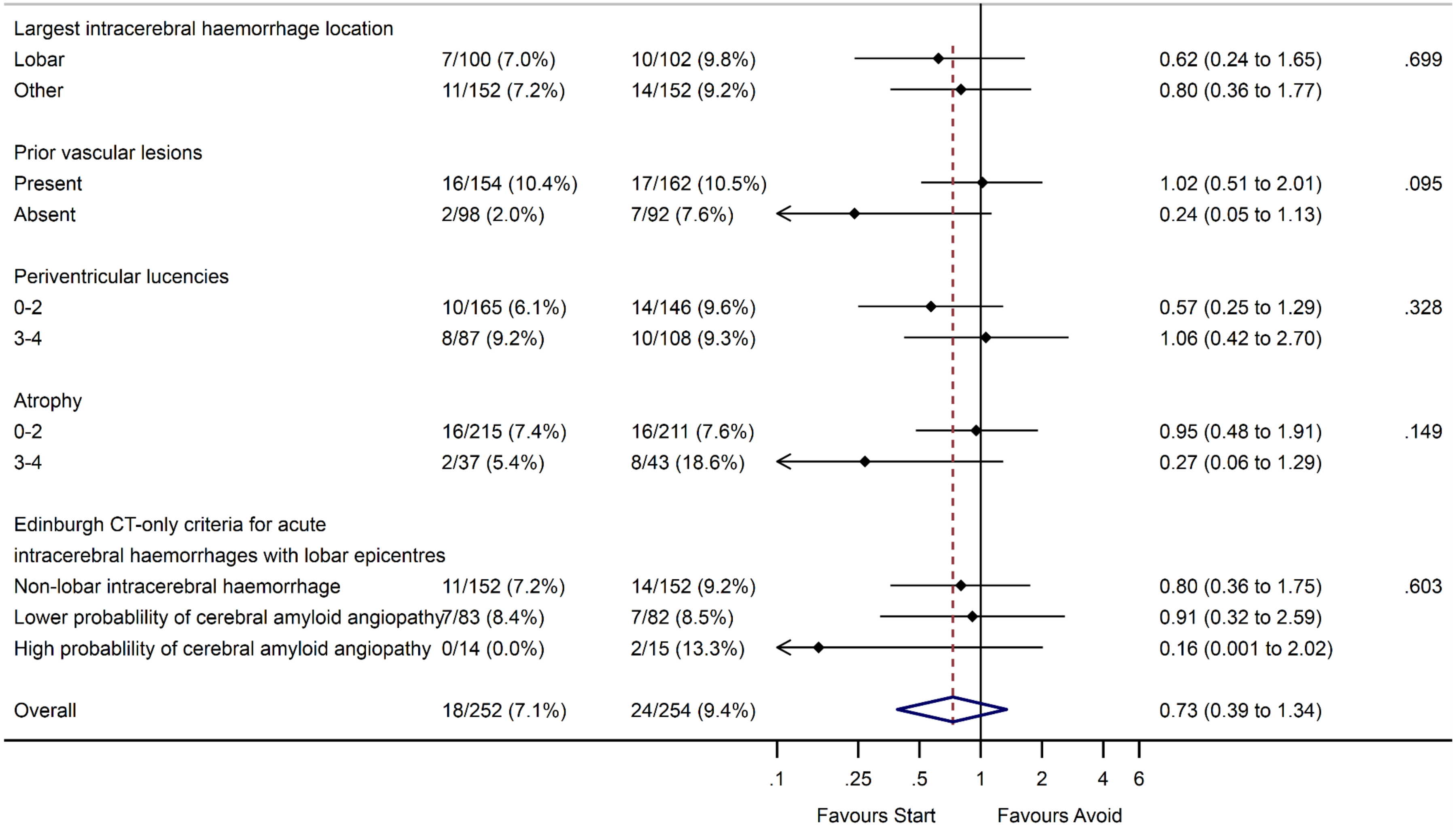

Association between cerebral microbleeds on MRI and first recurrent symptomatic intracerebral haemorrhage (the primary outcome) in 235 participants who had a brain MRI sequence of sufficient quality to rate cerebral microbleeds

Cox proportional hazards regression models	Adjusted hazard ratio (95% CI)	Adjusted p-value
**Cerebral microbleed presence (2 or more versus 0 or 1)**		
Cerebral microbleed presence (2 or more versus 0 or 1)	3.62 (1.34 to 9.79)	0.011
Start versus avoid antiplatelet therapy	0.49 (0.18 to 1.32)	0.16
Lobar versus non-lobar intracerebral haemorrhage location	0.78 (0.30 to 2.02)	0.61
0-30 days versus >30 days since intracerebral haemorrhage onset	1.08 (0.37 to 3.10)	0.89
Intended prescription: aspirin versus other	0.90 (0.34 to 2.33)	0.82
<70 years versus 70 years or more at randomization	0.62 (0.17 to 2.23)	0.47
Predicted probability of a good outcome at six months: <0.15 versus 0.15 or more	2.58 (0.88 to 7.54)	0.084
**Cerebral microbleed burden (linear trend of 0 or 1, 2–4, and 5 or more)**		
Cerebral microbleed burden (linear trend of 0 or 1, 2–4, and 5 or more)	1.99 (1.20 to 3.31)	0.0077
Start versus avoid antiplatelet therapy	0.53 (0.20 to 1.42)	0.21
Lobar versus non-lobar intracerebral haemorrhage location	0.76 (0.29 to 1.97)	0.57
0-30 days versus >30 days since intracerebral haemorrhage onset	1.11 (0.38 to 3.19)	0.85
Intended prescription: aspirin versus other	0.83 (0.32 to 2.16)	0.70
<70 years versus 70 years or more at randomization	0.69 (0.19 to 2.45)	0.56
Predicted probability of a good outcome at six months: <0.15 versus 0.15 or more	2.80 (0.95 to 8.22)	0.061

Association between cerebral microbleeds on MRI and first ischaemic stroke (the secondary outcome) in 235 participants who had a brain MRI sequence of sufficient quality to rate cerebral microbleeds

Cox proportional hazards regression models	Adjusted hazard ratio (95% CI)	Adjusted p-value
**Cerebral microbleed presence (2 or more versus 0 or 1)**		
Cerebral microbleed presence (2 or more versus 0 or 1)	1.92 (0.83 to 4.46)	0.13
Start versus avoid antiplatelet therapy	0.70 (0.30 to 1.65)	0.42
Lobar versus non-lobar intracerebral haemorrhage location	0.90 (0.38 to 2.13)	0.81
0-30 days versus >30 days since intracerebral haemorrhage onset	1.34 (0.49 to 3.65)	0.57
Intended prescription: aspirin versus other	2.48 (0.91 to 6.81)	0.077
<70 years versus 70 years or more at randomization	1.31 (0.51 to 3.36)	0.57
Predicted probability of a good outcome at six months: <0.15 versus 0.15 or more	0.42 (0.09 to 1.94)	0.26
**Cerebral microbleed burden (linear trend of 0 or 1, 2–4, and 5 or more)**		
Cerebral microbleed burden (linear trend of 0 or 1, 2–4, and 5 or more)	1.62 (1.03 to 2.55)	0.038
Start versus avoid antiplatelet therapy	0.69 (0.29 to 1.63)	0.40
Lobar versus non-lobar intracerebral haemorrhage location	0.86 (0.36 to 2.04)	0.73
0-30 days versus >30 days since intracerebral haemorrhage onset	1.36 (0.50 to 3.72)	0.55
Intended prescription: aspirin versus other	2.47 (0.90 to 6.78)	0.078
<70 years versus 70 years or more at randomization	1.45 (0.56 to 3.73)	0.44
Predicted probability of a good outcome at six months: <0.15 versus 0.15 or more	0.41 (0.09 to 1.92)	0.26

Primary and exploratory sub-group analyses of the risk of first ischaemic stroke (the secondary outcome) by MRI biomarkers

Subgroup	Start antiplatelet drugs [Events/N(%)]	Avoid antiplatelet drugs [Events/N(%)]	Adjusted Hazard Ratio (95% CI)	Test of interaction p-value
Largest intracerebral haemorrhage				
Lobar	3/52 (5.8%)	5/56 (8.9%)	0.53 (0.12 to 2.22)	.577
Other	6/70 (8.6%)	8/76 (10.5%)	0.88 (0.30 to 2.54)	
Old ischaemic lesions				
None	5/72 (6.9%)	6/74 (8.1%)	1.03 (0.31 to 3.43)	.418
One or more	4/50 (8.0%)	7/58 (12.1%)	0.49 (0.14 to 1.75)	
Old haemorrhagic lesions				
None	8/110 (7.3%)	12/112 (10.7%)	0.66 (0.27 to 1.62)	.507
One or more	1/12 (8.3%)	1/20 (5.0%)	1.79 (0.11 to 29.69)	
Superficial siderosis				
Focal/Disseminated	3/27 (11.1%)	2/33 (6.1%)	2.04 (0.34 to 12.28)	.201
None	6/95 (6.3%)	11/99 (11.1%)	0.53 (0.20 to 1.45)	
White matter hyperintentsities				
0-2	1/39 (2.6%)	5/43 (11.6%)	0.19 (0.02 to 1.69)	.157
3-6	8/83 (9.6%)	8/89 (9.0%)	1.10 (0.40 to 3.01)	
Atrophy				
0-2	3/76 (3.9%)	5/71 (7.0%)	0.57 (0.13 to 2.39)	.507
3-4	6/46 (13.0%)	8/61 (13.1%)	1.05 (0.35 to 3.11)	
Cerebral microbleeds				
Presence				
0 or 1	3/66 (4.5%)	8/76 (10.5%)	0.39 (0.10 to 1.46)	.265
2 or more	6/48 (12.5%)	5/45 (11.1%)	1.07 (0.32 to 3.62)	
Number				
0 or 1	3/66 (4.5%)	8/76 (10.5%)	0.42 (0.10 to 1.38)	.467
2 to 4	1/16 (6.3%)	0/15 (0.0%)	3.76 (0.18 to 575.1)	
5 or more	5/32 (15.6%)	5/30 (16.7%)	0.86 (0.25 to 2.95)	
Location				
Strictly lobar	0/7 (0.0%)	1/13 (7.7%)	0.51 (0.003 to 10.18)	.692
Other	6/41 (14.6%)	4/32 (12.5%)	1.13 (0.30 to 4.56)	
Modified Boston CAA criteria				
Probable cerebral amyloid angiopathy	2/19 (10.5%)	3/28 (10.7%)	0.86 (0.14 to 5.34)	.89
Possible cerebral amyloid angiopathy	1/14 (7.1%)	2/16 (12.5%)	0.41 (0.03 to 4.75)	
Neither possible nor probable CAA	6/81 (7.4%)	8/77 (10.4%)	0.69 (0.24 to 2.05)	
Overall	9/122 (7.4%)	13/132 (9.8%)	0.73 (0.31 to 1.71)	

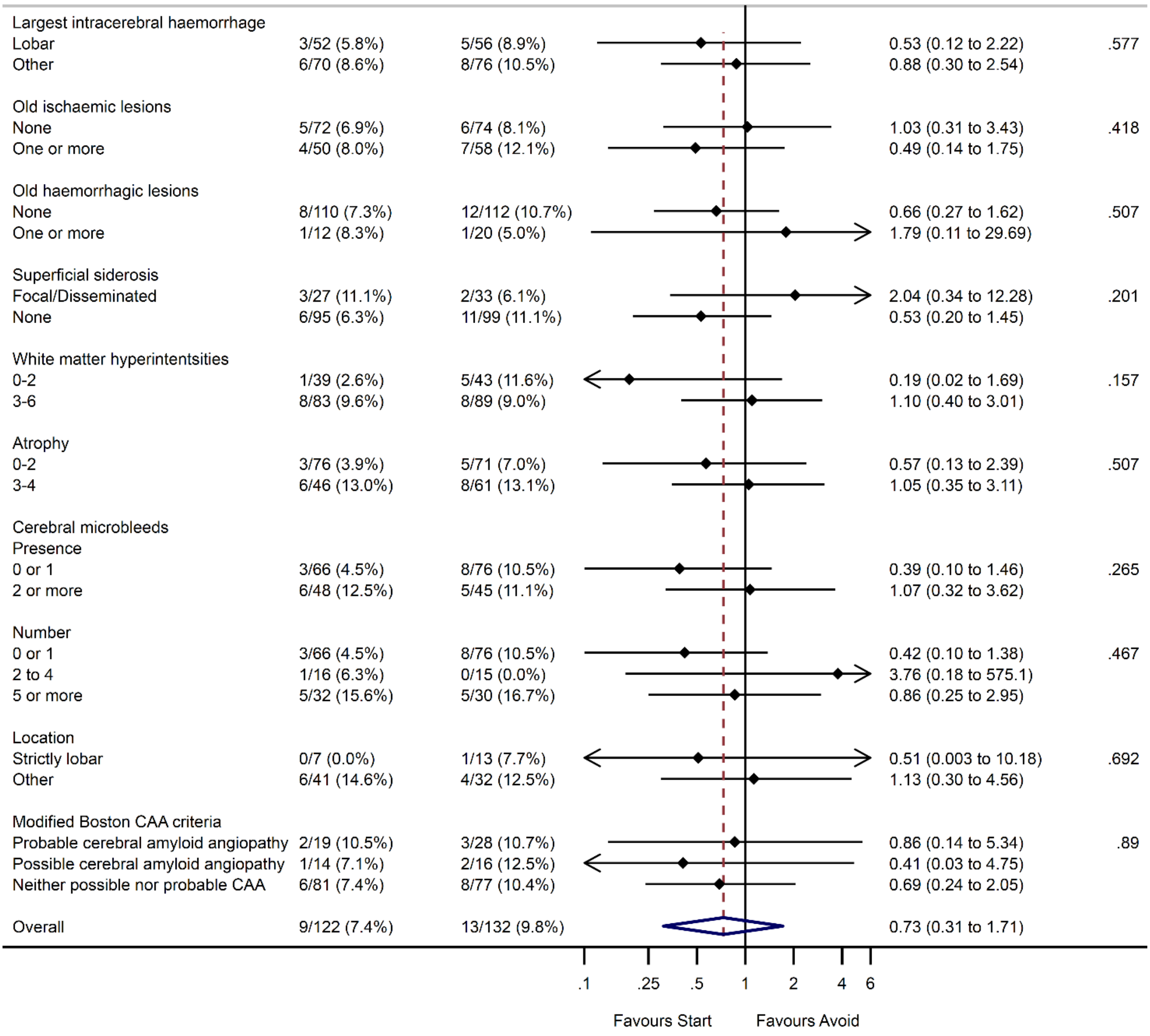

Supplement: Supplementary appendix [file mmc1.pdf]
